# Cancer risk based on alcohol consumption levels: a comprehensive systematic review and meta-analysis

**DOI:** 10.4178/epih.e2023092

**Published:** 2023-10-16

**Authors:** Seunghee Jun, Hyunjin Park, Ui-Jeong Kim, Eun Jeong Choi, Hye Ah Lee, Bomi Park, Soon Young Lee, Sun Ha Jee, Hyesook Park

**Affiliations:** 1 Department of Preventive Medicine, Ewha Womans University College of Medicine, Seoul, Korea; 2 Graduate Program in System Health Science and Engineering, Ewha Womans University College of Medicine, Seoul, Korea; 3 National Cancer Control Institute, National Cancer Center, Goyang, Korea; 4 Clinical Trial Center, Ewha Womans University Mokdong Hospital, Seoul, Korea; 5 Department of Preventive Medicine, Chung-Ang University College of Medicine, Seoul, Korea; 6 Department of Preventive Medicine and Public Health, Ajou University School of Medicine, Suwon, Korea; 7 Institute for Health Promotion, Department of Epidemiology and Health Promotion, Graduate School of Public Health, Yonsei University, Seoul, Korea

**Keywords:** Systematic review, Meta-analysis, Alcohol drinking, Neoplasms, Cohort studies

## Abstract

**OBJECTIVES:**

Alcohol consumption is a well-established risk factor for cancer. Despite extensive research into the relationship between alcohol consumption and cancer risk, the effect of light alcohol consumption on cancer risk remains a topic of debate. To contribute to this discourse, we conducted a comprehensive systematic review and meta-analysis.

**METHODS:**

Our systematic review aimed to investigate the associations between different levels of alcohol consumption and the risk of several cancer types. We focused on analyzing prospective associations using data from 139 cohort studies. Among them, 106 studies were included in the meta-analysis after a quantitative synthesis.

**RESULTS:**

Our analysis did not find a significant association between light alcohol consumption and all-cause cancer risk (relative risk, 1.02; 95% confidence interval, 0.99 to 1.04), but we observed a dose-response relationship. Light alcohol consumption was significantly associated with higher risks of esophageal, colorectal, and breast cancers. Light to moderate drinking was associated with elevated risks of esophageal, colorectal, laryngeal, and breast cancers. Heavy drinking was also found to contribute to the risk of stomach, liver, pancreas, and prostate cancers, thereby increasing the risk of almost all types of cancer. Additionally, females generally had lower cancer risks compared to males.

**CONCLUSIONS:**

Our findings highlight that cancer risks extend beyond heavy alcohol consumption to include light alcohol consumption as well. These findings suggest that there is no safe level of alcohol consumption associated with cancer risk. Our results underscore the importance of public health interventions addressing alcohol consumption to mitigate cancer risks.

## GRAPHICAL ABSTRACT

[Fig f4-epih-45-e2023092]

## INTRODUCTION

Cancer is the second leading cause of death globally, accounting for almost 10 million deaths in 2020 [1]. Alcohol consumption causes a range of diseases, including cancer [2]. In 1988, the International Agency for Research on Cancer (IARC) classified alcohol as a Group 1 human carcinogen [3]. The IARC also lists alcohol as a cause of breast, colorectal, laryngeal, liver, esophageal, oral, and pharyngeal cancers [4,5]. According to the Global Burden of Cancer in 2020 Attributable to Alcohol Consumption study, approximately 741,300 cancer cases (95% uncertainty interval [UI], 558,500 to 951,200; population-attributable fraction, 4.1% [95% UI, 3.1 to 5.3]) were attributable to alcohol consumption [6].

The association between alcohol consumption and the risk of cancer death follows a J-shaped pattern, where heavy drinkers exhibit a higher cancer mortality rate than moderate drinkers [7,8]. Cohort studies have shown that even a small amount of alcohol consumption is associated with a heightened risk of cancer and death [9,10]. Furthermore, a meta-analysis reported that light alcohol consumption (1 drink per day, < 12 g alcohol) was linked to increased risks of esophageal (30%), oropharyngeal (17%), liver (8%), colon (7%), and breast (5%) cancers [11]. Furthermore, among female, light alcohol consumption was associated with 20% excess risks of breast and colorectal cancers [12].

Given the evidence linking alcohol consumption with cancer, various countries have established guidelines on alcohol intake [13,14]. For instance, the IARC’s third edition of the European Code Against Cancer (ECAC) recommended a limit of “no more than two drinks a day for male and one drink per day for female” [15]. The fourth edition now recommends, “If you drink alcohol of any type, limit your intake. Not drinking alcohol is better for cancer prevention” [16]. Korea has similarly revised cancer prevention guidelines, shifting from ‘limiting alcohol to 1-2 drinks a day’ to virtually abstaining from drinking [17].

Critics have argued that the meta-analysis by Bagnardi et al. [11], which was central to the fourth edition of the ECAC guidelines [16], may have produced inaccurate recommendations [18]. In particular, it has been pointed out that this study failed to take into consideration the differences between case-control and cohort study design, which resulted in an incorrect conclusion being drawn from a combination of findings from both study types [18,19]. The study of Choi et al. [19] supported the link between light alcohol consumption and an increased risk of cancer. However, it should be noted that their study focused only on light drinking patterns and therefore could not determine the effects of varying alcohol consumption levels. As most existing research has focused on specific levels of alcohol consumption or specific cancer types, there is still a lack of studies establishing the risk relationship between different drinking levels and various cancer types.

Our study sought to investigate the association between light to heavy alcohol intake and cancer risk, unlike many studies that have focused solely on specific alcohol consumption levels. We also concentrated on a variety of cancer types identified as alcohol-related carcinomas by the IARC [20]. To ensure our studies drew from the most recent evidence, we integrated data from the Korean Cancer Prevention Study-II and the Korean Genome Epidemiology Study Biobank (Pooled Korean Biobank) [21]. This approach allowed us to encompass a broader range of literature than previous meta-analyses, thereby enabling us to present up-to-date findings.

## MATERIALS AND METHODS

**Figure 1.**
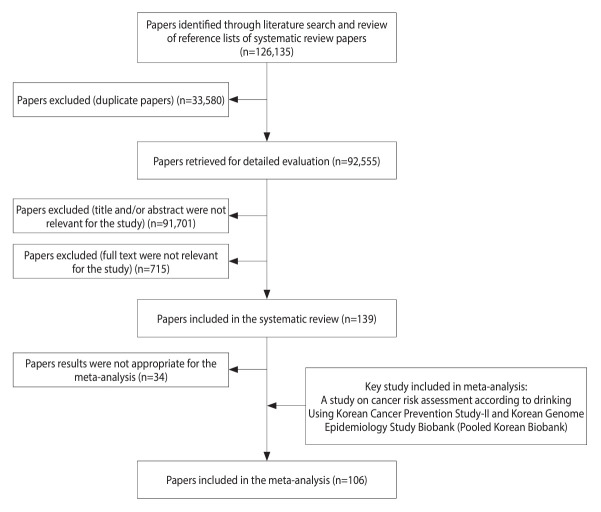
Flow chart of study selection for inclusion in the meta-analysis.

We conducted the study following the Preferred Reporting Items for Systematic Review and Meta-Analysis (PRISMA) guidelines (Figure 1). A systematic literature search was conducted using 5 databases (Embase, Cochrane Library, PubMed, Scopus, and Web of Science) to identify original articles published up to July 2021. The search was performed using relevant keywords such as “alcohol,” “alcohol drinking,” “neoplasm,” “cancer,” “carcinoma,” and “cohort.” Only studies published in English were considered. In addition to the database search, the references of the retrieved articles were manually screened to identify any additional relevant studies.

Two authors (SJ and HjP) independently reviewed the titles and abstracts of the retrieved articles based on pre-set eligibility criteria. The full texts of the remaining articles after the initial screening were assessed by both researchers independently for final inclusion. Any inconsistencies between the 2 researchers were resolved through a consensus meeting with other authors (HAL BP, SYL and HsP).

The inclusion criteria for the meta-analysis were as follows: (1) studies focusing on esophageal, stomach, liver, pancreatic, colorectal, larynx, lung, thyroid, breast, and prostate cancers; (2) original cohort studies (excluding abstracts, letters, reviews, and meta-analyses); (3) studies reporting quantitative findings regarding the relationship between alcohol consumption and the risk of cancer as hazard ratios (HRs), relative risks (RRs), or odds ratios (ORs) for alcohol drinkers compared to non-drinkers or occasional drinkers; (4) studies providing the standard error or confidence interval (CI) of the risk estimate or sufficient data for their calculation; and (5) studies with the full text accessible. If multiple articles reported results from the same study, the most recent or complete article was included. Studies that evaluated specific types of alcoholic beverages only were excluded to avoid potential confounding.

Data extraction included information such as first author, year of publication, country, follow-up duration, cohort size and cases, sex, alcohol consumption level, OR/RR/HR and 95% CI, confounders considered, and outcomes (cancer incidence or death). If available, data were evaluated by sex. The methodological quality of the included studies was assessed independently by 2 authors (SJ and HjP) using the Newcastle-Ottawa Scale (NOS), with a higher score indicating higher methodological quality (NOS score ≥ 7 considered high-quality). Any disagreements or uncertainties were resolved through discussion to reach a consensus.

For the purpose of analysis, alcohol consumption was categorized into 4 levels: light (0.01-12.4 g/day), light to moderate (12.5-24.9 g/day), moderate to high (25.0-49.9 g/day), and heavy (≥ 50.0 g/day). Various units of alcohol consumption (grams, milliliters, ounces, or number of drinks) reported in the studies were converted to grams per day using the following conversion factors: 0.8 g/mL, 28 g/oz, and 12 g/glass [22]. In studies that provided cancer risk based on categories of alcohol intake, the median value of each category was used for analysis [23].

A meta-analysis was performed to estimate the pooled RR of cancer for each alcohol consumption level, except for laryngeal cancer due to insufficient data. Since cancer is a rare disease, the OR, RR, and HR were assumed to be comparable estimates of the RR. The analysis included risk estimates that were adjusted for confounding factors, and if adjusted values were unavailable, crude values were used. We computed pooled RR estimates for the reported cancer risk compared to non-drinkers by alcohol consumption level. All analyses were performed without distinguishing between incident cancer and death.

Forest plots were used to identify heterogeneity between studies, and the degree of heterogeneity was quantified using the I^2^ statistic, with low (< 25%), medium (25 to ≤ 75%), and high (> 75%) [24] levels of heterogeneity considered. A fixed-effects model was used in most cases; however, a random-effects model was used if there was high heterogeneity between studies. To assess the dose-response association between alcohol consumption and cancer risk, a dose-response meta-analysis was conducted. The linearity and non-linearity of associations were evaluated [25]. Publication bias was evaluated using funnel plots and Egger’s regression asymmetry test [26].

The statistical analysis was carried out using R studio version 4.2.1 and the “metafor” package in R software (https://cran.r-project.org/). The results of 2-sided tests were considered statistically significant when p< 0.05.

### Ethics statement

This study is not required to ethical approval since it was based on published articles.

## RESULTS

We conducted a comprehensive study selection process, which is presented in Figure 1. Initially, we identified a total of 126,135 articles, including systematic review papers, through a literature search. After excluding 33,580 duplicates articles, we reviewed the titles and abstracts of the remaining 92,555 articles, resulting in the exclusion of 91,701 irrelevant articles. After reviewing the full text of 854 articles, 715 were excluded because they had insufficient data (n=169), were published in a language other than English (n=8), were gray literature (n=27), had an inaccessible full text (n=150), dealt with cancer types other than those under investigation (n = 105), and had case–control or other study designs (n=256). The systematic review included a total of 139 papers. Among these, 34 articles were considered unsuitable for assessing cancer risk associated with alcohol consumption and were subsequently excluded. The final meta-analysis comprised 106 articles, which included an additional key study for a comprehensive analysis. The reference list categorized by cancer type is shown in Supplementary Marterials 1 and 2 provides a summary of confounding factors stratified by cancer type, based on the selected literature.

Table 1 summarizes the characteristics of the studies included in the meta-analysis by cancer type, excluding the key study. Of the included articles, 73 were published before 2010, while 32 were published after 2010. These studies were conducted in the Americas (n=43), Asia (n=32), and Europe or Australia (n=30). The cohort size ranged from 2,682 to 6,568,561 for male and 1,954 to 6,568,561 for female, while the number of cases ranged from 11 to 64,476 for male and 3 to 41,315 for female. Some articles did not report cohort size by sex. The follow-up period varied from 4 to 47 years. It should be noted that articles were counted more than once if they investigated multiple cancer types or reported results from multiple cohort studies. Further details on the characteristics of the studies can be found in Supplementary Material 1.

**Figure 2.**
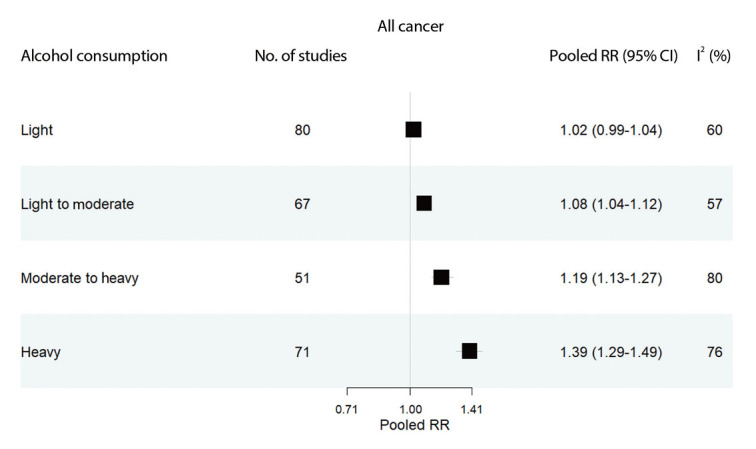
Pooled relative risks estimated by all cancer (esophageal; stomach; liver; pancreatic; colorectal; laryngeal; lung; thyroid; prostate; and breast) and alcohol consumption levels. The range of alcohol consumption levels was divided into light (0.01-12.4 g/day), light to moderate (12.5-24.9 g/day), moderate to heavy (25.0-49.9 g/day), and heavy (≥50.0 g/day). RR, relative risk; CI, confidence intervals; I^2^, indicates heterogeneity.

All studies included in the meta-analysis analyzed cancer risk according to alcohol consumption level, regardless of the specific cancer type. Light alcohol consumption was not significantly associated with all-cause cancer risk (RR, 1.02; 95% CI, 0.99 to 1.04). However, alcohol consumption levels from light to moderate (RR, 1.08; 95% CI, 1.04 to 1.12), moderate to heavy (RR, 1.19; 95% CI, 1.13 to 1.27), and heavy (RR, 1.39; 95% CI, 1.29 to 1.49) were each significantly associated with an increased risk of cancer. These findings confirm a dose-response relationship, where higher levels of alcohol consumption are linked to greater cancer risk (Figure 2). This is also supported by the linearity results of the dose-response meta-analysis (p for linearity < 0.001; data not shown). Additionally, females exhibited lower risks of all cancer types than males, and both males and females demonstrated a dose–response relationship between alcohol consumption and the risk of all cancer types (Supplementary Material 3).

We detected evidence of publication bias for heavy drinking (Egger’s test, p=0.01), as indicated by asymmetry in the funnel plot. However, it should be noted that for esophageal cancer, the calculation of a large risk value may have influenced the symmetry of the plot. There was no evidence of publication bias for other alcohol consumption levels (Supplementary Material 4).

**Figure 3.**
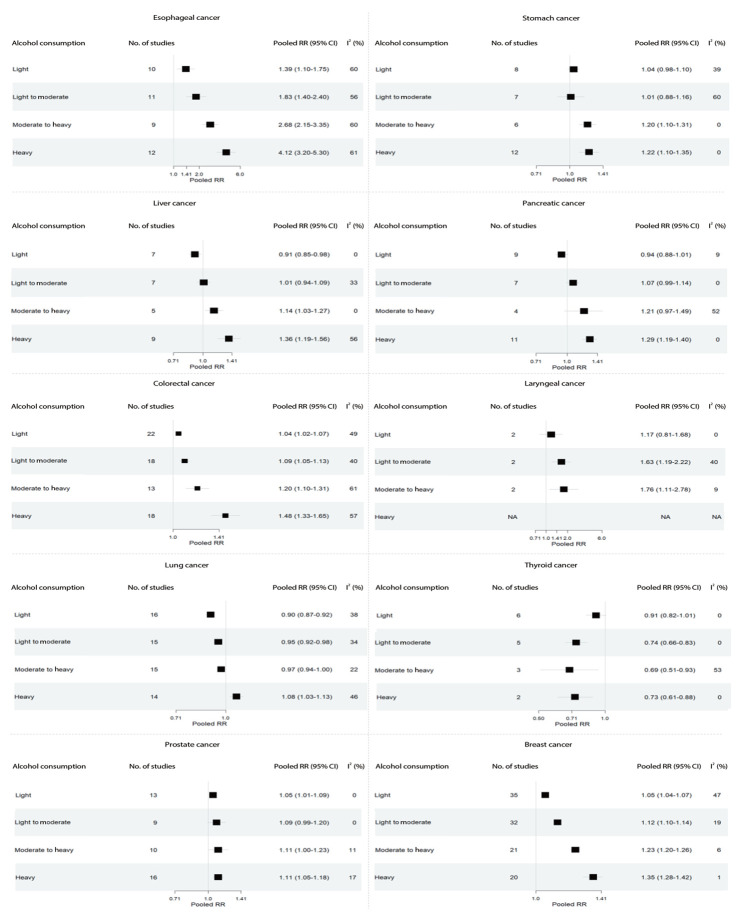
Pooled RR estimated by cancer types and alcohol consumption levels. The range of alcohol consumption levels was divided into light (0.01-12.4 g/day), light to moderate (12.5-24.9 g/day), moderate to heavy (25.0-49.9 g/day), and heavy (≥50.0 g/day). RR, relative risk; CI, confidence intervals; I^2^, indicates heterogeneity; NA, not available.

Figure 3 shows the pooled RRs for each cancer type according to alcohol consumption level. Except for thyroid cancer, all other cancers showed a dose–response relationship with alcohol consumption, where the RR increased as the level of alcohol consumption increased. Heavy alcohol consumption was significantly associated with an elevated risk of all cancer types except thyroid cancer. Furthermore, light alcohol consumption was significantly associated with the risk of esophageal cancer (RR, 1.39; 95% CI, 1.10 to 1.75), colorectal cancer (RR, 1.04; 95% CI, 1.02 to 1.07), prostate cancer in male (RR, 1.05; 95% CI, 1.01 to 1.09), and breast cancer in female (RR, 1.05; 95% CI, 1.04 to 1.07). For light to moderate alcohol consumption, increased risks were evident for esophageal cancer (RR, 1.83; 95% CI, 1.40 to 2.40), colorectal cancer (RR, 1.09; 95% CI, 1.05 to 1.13), laryngeal cancer (RR, 1.63; 95% CI, 1.19 to 2.22), and breast cancer in female (RR, 1.12; 95% CI, 1.10 to 1.14). As the level of alcohol consumption increased to moderate to heavy, there were significant elevations in risk for esophageal (RR, 2.68; 95% CI, 2.15 to 3.35), stomach (RR, 1.20; 95% CI, 1.10 to 1.31), liver (RR, 1.14; 95% CI, 1.03 to 1.27), colorectal (RR, 1.20; 95% CI, 1.10 to 1.31), laryngeal (RR, 1.76; 95% CI, 1.11 to 2.78), prostate (RR, 1.11; 95% CI, 1.00 to 1.23), and breast cancers (RR, 1.23; 95% CI, 1.20 to 1.26). Heavy drinking was associated with an elevated risk for nearly all the evaluated cancers, with the exception of thyroid and laryngeal cancers.

The results of analysis stratified by sex are shown in Table 2. Light alcohol consumption was significantly associated with the risk of esophageal cancer in male and female (male: RR, 1.65, 95% CI, 1.36 to 1.99; female: RR, 1.17, 95% CI, 1.00 to 1.37). Moreover, the risk of colorectal cancer in male was associated with light alcohol consumption (RR, 1.16; 95% CI, 1.04 to 1.28). At moderate to heavy consumption levels, both male and female demonstrated heightened risks of cancer, with the exception of liver cancer, lung cancer, and pancreatic cancer in female. Heavy levels of alcohol consumption were found to elevate the risk of cancer for both male and female, except for colorectal and lung cancers in female. There was no evidence for publication bias for Esophageal, colorectal, lung, prostate, or breast cancer (except for light to moderate drinking) (Supplementary Material 5). Publication bias was not analyzed for other cancer types due to the limited number of studies [27].

## DISCUSSION

This systematic review and meta-analysis aimed to examine the association between levels of alcohol consumption and cancer-specific risk. A total of 139 papers were included in the systematic review, and 106 papers were included in the subsequent meta-analysis. The analysis revealed a dose–response relationship, indicating that as alcohol consumption levels increased, the risk of cancer also increased for most cancer types. However, the impact of light alcohol consumption varied across cancer types. Significant associations were found between light alcohol consumption and esophageal, colorectal, prostate (in male), and breast (in female) cancers. Light to moderate drinking significantly elevated the risk of specific cancers, including esophageal, colorectal, laryngeal, and breast cancer (in female). Moderate and heavy drinking further escalated the risk, including for additional cancer types, including stomach, liver, and pancreatic cancers. Heavy drinking was associated with almost all evaluated cancers except for thyroid and laryngeal cancers. Furthermore, female generally had lower risks of all cancer types than male.

Our findings underscore the importance of introducing public health interventions and educational programs aimed at raising awareness about the heightened cancer risks linked to even modest levels of alcohol consumption. This is especially relevant concerning esophageal, colorectal, prostate (for male), and breast cancers (for female). As safe drinking limits for cancer risk have not been identified, it is crucial to develop appropriate interventions to address drinking behavior. The 2021 Korean National Cancer Prevention Awareness and Practice Survey showed that the guideline to “avoid alcohol consumption” was challenging for many respondents [28]. Furthermore, the percentage of respondents who adhered to the guideline by abstaining from alcohol declined from 76.2% in 2007 to 47.7% in 2021 [28]. From 2019 to 2020, the drinking rate of adults aged 19 and older in Korea was 74.8%, higher than the global average drinking rate of 43% (≥15 years) [29,30]. Although monthly drinking rates in Korea have decreased, over half of the population still consumes alcohol at least once a month [31]. Notably, around a quarter of these individuals consume 1 drink to 2 drinks per session [32]. Our findings showed that even light alcohol consumption, defined as less than 1 drink, was associated with increased risks of esophageal, colorectal, prostate (in male), and breast (in female) cancers. Apart from esophageal cancer, colorectal, prostate, and breast cancers rank among the top 5 most common cancers in Korea [33]. Given Korea’s high alcohol consumption rates, adhering to drinking guidelines might be an effective strategy to reduce the incidence of these cancers.

Our study results align with previous meta-analyses [11,19,34], indicating a dose–response relationship between alcohol consumption level and cancer risk. This consistency is further supported by a previous cohort study conducted in Korean male, which followed participants for 10.5 years and included repeated measurements of alcohol consumption [35]. Compared to non-drinkers, mild drinkers (< 15 g/day) exhibited a 4% increased risk, while medium-risk drinkers (15-30 g/day) and high-risk drinkers (≥ 30 g/day) experienced 14% and 28% increased risks, respectively [36].

In this study, light alcohol consumption was associated with a significantly higher risk of colorectal cancer and prostate cancer, with a significantly higher risk of colorectal cancer, especially in male. This finding is consistent with other previous studies. A previous study showed a significant dose–response relationship between low levels of alcohol consumption (> 1.33 g and < 25 g/day) and the prostate cancer risk [37]. In addition, Choi et al. [19] reported that light alcohol consumption slightly increased the incidence of colorectal cancer in males. A possible mechanism to explain this is that folic acid and acetaldehyde, which are present in alcoholic beverages, have been reported to promote colorectal carcinogenesis [38].

Our study revealed a strong association between esophageal cancer and alcohol consumption across all levels. This finding aligns with previous research by Bagnardi et al. [11], whose meta-analysis demonstrating a 30% increased risk of esophageal cancer, particularly squamous cell carcinoma, among individuals with light alcohol consumption (≤ 12.5 g/day; ≤ 1 drink/day). Notably, this risk was prominently observed in Asians (RR, 1.49; 95% CI, 1.12 to 1.98) [11], potentially due to genetic variations in ethanol metabolism [39]. Furthermore, another meta-analysis reported a significant increase in esophageal cancer risk at high alcohol consumption (100 g/day; RR, 4.23; 95% CI, 3.91 to 4.59) [40].

Our study found a strong association between breast cancer and alcohol consumption across all levels. Consistent with previous studies [41,42], even low levels of alcohol consumption were linked to an increased incidence of breast cancer. In a meta-analysis [42], it was observed that each additional 10 g of alcohol consumed per day increased the risk of breast cancer in female by 7.1% (95% CI, 5.5 to 8.7). Similarly, our study revealed that higher levels of alcohol consumption were associated with a higher risk of breast cancer in females. Notably, even very light alcohol consumption, defined as less than 0.5 drinks per day, increased the risk of breast cancer (RR, 1.04; 95% CI, 1.01 to 1.07) [19], indicating that there is no safe level of alcohol consumption in terms of increased risk of breast cancer. This heightened risk can be attributed to alcohol’s impact on the mammary gland, including elevating estrogen and insulin-like growth factor concentrations, altering structural development, and stimulating cell proliferation [43].

No significant association was found between thyroid cancer and any level of alcohol consumption, suggesting a potential protective role for alcohol. This finding is consistent with the results of 2 previous meta-analyses [19,44]. Alcohol consumption may play a preventive role in the development of thyroid cancer by reducing thyroid-stimulating hormone levels [45]. Additionally, alcohol might exert a direct toxic effect on thyroid cells, leading to a reduction in thyroid volume and a decreased risk of thyroid cancer [46]. However, due to the limited number of studies available on thyroid cancer, further research is warranted to confirm these findings.

In contrast, alcohol consumption has been identified as a risk factor for laryngeal cancer [11,19,47]. However, the available studies were insufficient to assess the association between heavy alcohol consumption and laryngeal cancer. Nonetheless, a moderate level of alcohol consumption was found to increase the risk of laryngeal cancer.

This study has several limitations that warrant acknowledgment. First, while a thorough assessment of literature quality indicated a low overall risk of bias, caution is needed in interpreting the findings because of the potential for bias and confounding effects. Second, variations in how different studies defined drinking levels could have introduced inconsistencies. For example, some studies did not distinguish between non-drinkers and former drinkers. This could have impacted the accuracy of the estimated effect size. Third, the type of alcoholic beverage (e.g., wine, beer, liquor) was not taken into account, although it is known that different types of alcohol may have varying effects on health outcomes. Fourth, since alcohol consumption is often self-reported, there is a possibility of recall bias influencing the effect size. Heavy drinkers might underreport their consumption, or individuals may underestimate their alcohol intake due to memory lapses [48,49]. Additionally, misclassifications and errors may arise from the use of different survey tools across the included studies. To address the issue of heterogeneity, a random-effects model was employed when high between-study heterogeneity was observed [27]. Lastly, we did not perform a stratified analysis of confounding factors to pinpoint potential sources of heterogeneity, so the results should be interpreted with caution.

Nonetheless, our study has several notable strengths. Firstly, we incorporated the most recent literature available in our review [21], ensuring that our review reflects the most up-to-date findings compared to previous meta-analyses conducted on this topic. Secondly, while previous meta-analyses primarily focused on exploring the association between specific doses of alcohol consumption and cancer risk, our study took a broader approach by investigating the association between various levels of alcohol consumption and cancer risk. This comprehensive analysis provides a more nuanced understanding of the topic. Lastly, we specifically aimed to include only cohort studies in our analysis, which provide stronger evidence for causal association.

## CONCLUSION

Our findings support the proposal that higher levels of alcohol consumption are associated with an increased risk of cancer. Furthermore, even low levels of alcohol consumption have been found to elevate the risk of esophageal cancer, colorectal cancer, and breast cancer in females, as well as prostate and colorectal cancer in males. These findings suggest that there is no safe level of alcohol consumption in terms of cancer risk. In light of these findings, public health interventions, such as the strengthening of drinking guidelines, are necessary to mitigate the potential harm associated with alcohol consumption.

## Figures and Tables

**Figure f4-epih-45-e2023092:**
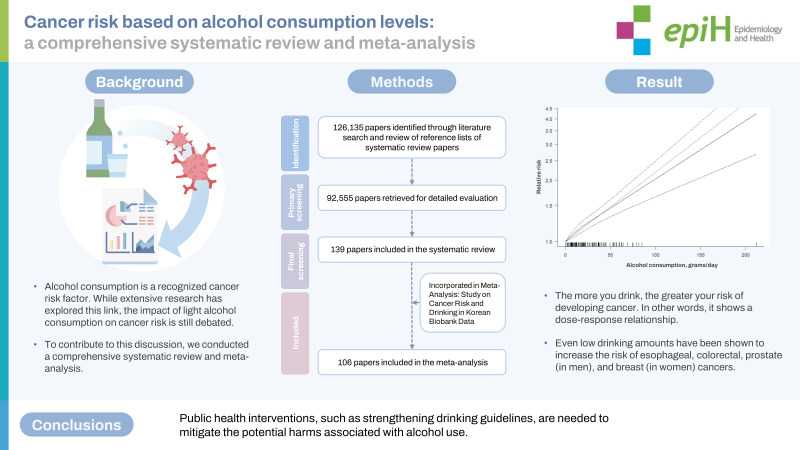


**Table 1.** Characteristics of studies included in the meta-analysis on cancer and alcohol consumption

Cancer type	No. of studies	Publication year		Country		Cohort size (range)		Case size (range)		Age at baseline, range (yr)	Year(s) of baseline, range	Outcome (no. of studies)		Duration of follow-up, range (yr)
		Year	No. of studies	Geographical area	No. of studies	Male	Female	Male	Female					
Esophageal cancer	14	Before 2010	7	Asia	12	2,696-919,199	3,595-1,280,296	11-42,756	3-773	40-80	1965-2004	Death	10	5-20.8
				Europe & Australia	1									
		After 2010	7	America	1							Incidence	4	
				Africa	0									
Stomach cancer	14	Before 2010	6	Asia	10	2,696-919,199	3,595-1,280,296	64-3,452	44-821	18-66.6	1965-2004	Death	6	5-46
				Europe & Australia	1									
		After 2010	8	America	3							Incidence	8	
				Africa	0									
Liver cancer	10	Before 2010	3	Asia	8	2,696-919,199	3,595-1,280,296	36-3,807	19-3,807	20-66.6	1965-2003	Death	7	5-20.8
				Europe & Australia	1									
		After 2010	7	America	1							Incidence	3	
				Africa	0									
Pancreatic cancer	16	Before 2010	7	Asia	5	2,696-919,199	3,595-1,280,296	15-3,443	14-3,404	30-84	1972-2008	Death	6	5-24
				Europe & Australia	5									
		After 2010	9	America	6							Incidence	10	
				Africa	0									
Colorectal cancer	27	Before 2010	14	Asia	13	2,682-5,105,889	3,595-3,654,706	26-64,476	16-36,569	18-75	1972-2005	Death	9	5-30
				Europe & Australia	7									
		After 2010	13	America	7							Incidence	18	
				Africa	0									
Laryngeal cancer	2	Before 2010	1	Asia	1	919,199	1,280,296	49	138	48.3-55.9	1996-2001	Death	1	5-7.2
				Europe & Australia	1									
		After 2010	1	America	0							Incidence	1	
				Africa	0									
Lung cancer	20	Before 2010	11	Asia	6	4,265-6,568,561	4,265-6,568,561	70-10,227	75-10,227	18-84	1965-2008	Death	10	5-32.8
				Europe & Australia	4									
		After 2010	9	America	10							Incidence	10	
				Africa	0									
Thyroid cancer	5	Before 2010	3	Asia	0	55,040-292,101	69,153-1,280,296	170-172	169-421	41-62.55	1978-2001	Death	0	7.2-17.8
				Europe & Australia	1									
		After 2010	2	America	4							Incidence	5	
				Africa	0									
Prostate cancer	21	Before 2010	14	Asia	3	3,775-919,199	-	46-17,227	-	18-84	1971-2008	Death	5	4-46
				Europe & Australia	5									
		After 2010	7	America	13							Incidence	16	
				Africa	0									
Breast cancer	36	Before 2010	21	Asia	3	-	1,954-1,280,296	-	51-41,315	18-84	1959-2001	Death	5	1.99-28.5
				Europe & Australia	13									
		After 2010	15	America	20							Incidence	31	
				Africa	0									

**Table 2.** Pooled relative risks by sex and cancer types^1^

Cancer type	Sex	Light		Light to moderate		Moderate to heavy			Heavy		
		No. of studies	RR (95% CI)	No. of studies	RR (95% CI)	No. of studies	RR (95% CI)		No. of studies	RR (95% CI)	
Esophageal cancer	Male	8	1.65 (1.36, 1.99)	8	1.88 (1.52, 2.34)	7	2.86 (2.45, 3.33)		11	3.94 (3.04, 5.10)	
	Female	2	1.17 (1.00, 1.37)	2	1.21 (1.02, 1.42)	-	-	-	-	-	-
Stomach cancer	Male	4	1.00 (0.92, 1.08)	4	1.06 (0.96, 1.17)	4	1.19 (1.07, 1.33)		7	1.17 (1.03, 1.33)	
	Female	3	0.87 (0.58, 1.31)	2	1.02 (0.56, 1.87)	-	-	-	-	-	-
Liver cancer	Male	2	0.93 (0.81, 1.06)	2	1.15 (0.65, 2.04)	2	1.12 (0.96, 1.31)		4	1.21 (1.12, 1.31)	
	Female	4	0.94 (0.75, 1.17)	3	1.25 (0.98, 1.57)	-	-	-	-	-	-
Pancreatic cancer	Male	6	0.99 (0.89, 1.11)	5	1.05 (0.94, 1.17)	3	1.36 (1.19, 1.57)		8	1.28 (1.16, 1.41)	
	Female	4	0.91 (0.82, 0.99)	3	1.07 (0.97, 1.18)	2	1.15 (0.98, 1.36)		3	1.36 (1.15, 1.62)	
Colorectal cancer	Male	13	1.16 (1.04, 1.28)	11	1.14 (1.09, 1.20)	8	1.29 (1.23, 1.34)		12	1.55 (1.46, 1.66)	
	Female	11	1.00 (0.97, 1.04)	10	1.01 (0.78, 1.30)	4	1.07 (1.00, 1.14)		5	1.09 (0.79, 1.50)	
Lung cancer	Male	9	0.89 (0.85, 0.92)	8	0.90 (0.86, 0.94)	9	0.97 (0.93, 1.01)		8	1.11 (1.03, 1.20)	
	Female	8	0.93 (0.79, 1.09)	7	1.04 (0.98, 1.10)	6	1.00 (0.91, 1.10)		3	0.89 (0.66, 1.21)	
Thyroid cancer^2^	Female	4	0.89 (0.77, 1.03)	3	0.67 (0.55, 0.82)	-	-	-	-	-	-

RR, relative risk; CI, confidence intervals.

1 The range of alcohol consumption levels was divided into light (0.01-12.4 g/day), light to moderate (12.5-24.9 g/day), moderate to heavy (25.0-49.9 g/day), and heavy (≥50.0 g/day).

2 Thyroid cancer (male) and laryngeal cancer were not presented because data from 1 or no study were obtained in this study, and information on sex-specific cancer incidence was insufficient.

## SUPPLEMENTARY MATERIALS

Supplement Material 1. Summary of studies characteristics by cancer types.

Supplement Material 2. Summary of confounding factors by cancer type in selected literature.

Supplement Material 3. All cancer and sex specific pooled RR estimates by meta-analysis.

Supplement Material 4. Funnel plots of the meta-analysis of studies examining the relationship between all cancer and alcohol consumption levels.

Supplement Material 5. Funnel plots of the meta-analysis of studies examining the relationship between cancer type and alcohol consumption levels.
