# Supplementary material for: Cancer risk based on alcohol consumption levels: a comprehensive systematic review and meta-analysis
Source: Epidemiol Health. 2023 Oct 16;45:e2023092. doi: 10.4178/epih.e2023092 (PMC10867516; doi:10.4178/epih.e2023092)
Supplement: Supplement Material 1. — Summary of studies characteristics by cancer types. [file epih-45-e2023092-Supplementary-1.docx]

Supplementary Material 1. Summary of studies characteristics by cancer types.

| No. | Study  (publication year) | Country | Cohort size | | Case size | | Age at baseline (y) | **Year(s) of baseline** | **Method of follow-up** | Outcome | Mean duration of follow-up year (y) | Ref. |
| --- | --- | --- | --- | --- | --- | --- | --- | --- | --- | --- | --- | --- |
|  |  |  | M | F | M | F |  |  |  |  |  |  |
| *Esophageal cancer* | | | | | | | | | | | | |
| 1 | Yi, S. W. (2010) | Korea | 2,696 | 3,595 | 19 | 3 | 66.6 | 1985 | Death certificates | death | 20.8 | [1] |
| 2 | Kim, M. K. (2010) | Korea | 919,199 | - | 213 | - | 48.3 | 2000 | National death certificate data | death | 5 | [2] |
| 3 | Yang, L. (2012) | China | 218,189 | - | 242 | - | 54.3 | 1990-1991 | China’s national Disease Surveillance Points | death | 15 | [3] |
| 4 | Shen, C. (2013) | Hong Kong | 22,680 | 44,140 | 69 | 46 | ≥65 | 1998-2001 | Death registration | death | 10.5 | [4] |
| 5 | Fan, Y. (2008) | China | 18,244 | - | 101 | - | 56.1 | 1986-1989 | Shanghai Cancer Registry, the Shanghai Municipal Vital Statistics Office | death/incidence | 20 | [5] |
| 6 | Kono, S. (1987) | Japan | 5,130 | - | 11 | - | 49 | 1965 | Death certificates | death | 19 | [6] |
| 7 | Yi, S. W. (2016) | Korea | 107,735 | - | 108 | - | 58.8 | 2004 | Death certificates | death | 6 | [7] |
| 8 | Yaegashi, Y. (2014) | Japan | 42,408 | - | 196 | - | 40-80 | 1988-1990 | Death certificates | death | 19 | [8] |
| 9 | Allen, N. E. (2009) | UK | - | 1,280,296 | - | 773 | 55.9 | 1996-2001 | National Health Service (NHS) Central Registers | incidence | 7.2 | [9] |
| 10 | Kinjo, Y. (1998) | Japan | 100,840 | 119,432 | 328 | 112 | 40-69 | 1965 | Vital statistics | death | 16 | [10] |
| 11 | Sakata, K. (2005) | Japan | 42,578 | - | 100 | - | 40-80 | 1988-1990 | Death certificates | death | 9 | [11] |
| 12 | Kimm, H. (2010) | Korea | 782,632 | - | 2,379 | - | 44.16 | 1992-1995 | National cancer registry data and hospitalization records, national death certificate data | death/incidence | 14 | [12] |
| 13 | Boffetta, P. (1990) | USA | 276,802 | - | 42,756 | - | 40-59 | 1959 | Death certificates | death | 12 | [13] |
| 14 | Ishiguro, S. (2009) | Japan | 44,970 | - | 215 | - | - | 1990-1993 | major local hospital and population based cancer registries | incidence | 11(~2004) | [14] |
| *Stomach cancer* | | | | | | | | | | | | |
| 1 | Yi, S. W. (2010) | Korea | 2,696 | 3,595 | 99 | 52 | 66.6 | 1985 | Death certificates | death | 20.8 | [1] |
| 2 | Kim, M. K. (2010) | Korea | 919,199 | 422,194 | 1,326 | 282 | 48.3 (M)/ 49 (F) | 2000 | National death certificate data | death | 5 | [2] |
| 3 | Jung, E. J. (2012) | Korea | 6,405 | 9,915 | 93 | | ≥20 | 1993 | National death certificate | death | 9.3 | [15] |
| 4 | Yang, L. (2012) | China | 218,189 | - | 323 | - | 54.3 | 1990-1991 | China’s national Disease Surveillance Points | death | 15 | [3] |
| 5 | Shen, C. (2013) | Hong Kong | 22,680 | 44,140 | 180 | 155 | ≥65 | 1998-2001 | Death registration | death | 10.5 | [4] |
| 6 | Kono, S. (1987) | Japan | 5,130 | - | 116 | - | 49 | 1965 | Death certificates | death | 19 | [6] |
| 7 | Ma, S. H. (2015) | Korea | 7,583 | 11,280 | 641 | 308 | 54.8 | 1993-2004 | Korean national cancer registry and the national death certificate | death/incidence | 8.25 | [16] |
| 8 | Allen, N. E. (2009) | UK | - | 1,280,296 | - | 821 | 55.9 | 1996-2001 | National Health Service (NHS) Central Registers | incidence | 7.2 | [9] |
| 9 | Sung, N. Y. (2007) | Korea | 669,570 | - | 3,452 | - | 44 | 1996 | Korean national cancer registry and the national death certificate | death/incidence | 6.5 | [17] |
| 10 | Stemmermann, G. N. (1990) | USA | 8,006 | - | 174 | - | - | 1940-1942 | Hospital records, death certificates, and the Hawaii Tumor Registry, a statewide cancer registry | incidence | 47 (~1988) | [18] |
| 11 | Moy, K. A. (2010) | China | 18,244 | - | 391 | - | 57.5 (cases) | 1986-1989 | Population based Shanghai Cancer Registry and Shanghai Municipal Vital Statistics Office databases | incidence | 20 | [19] |
| 12 | Klatsky, A. L. (2015) | USA | 55,040 | 69,153 | 403 | | 41 | 1978-1985 | KP Cancer Registry | incidence | 17.8 | [20] |
| 13 | Nomura, A. M. (1995) | Japan | 7,972 | - | 250 | - | - | 1965-1968 | Hawaii Tumor Registry | incidence | 26 | [21] |
| 14 | Galanis, D. J. (1998) | USA | 5,610 | 6,297 | 64 | 44 | ≥18 | 1975-1980 | Hawaii Tumor Registry | incidence | 14.8 | [22] |
| *Liver cancer* | | | | | | | | | | | | |
| 1 | Yi, S. W. (2010) | Korea | 2,696 | 3,595 | 36 | 19 | 66.6 | 1985 | Death certificates | death | 20.8 | [1] |
| 2 | Kim, M. K. (2010) | Korea | 919,199 | 422,194 | 1,506 | 174 | 48.3 (M)/ 49 (F) | 2000 | National death certificate data | death | 5 | [2] |
| 3 | Jung, E. J. (2012) | Korea | 6,405 | 9,915 | 85 | | ≥20 | 1993 | National death certificate data | death | 9.3 | [15] |
| 4 | Yang, L. (2012) | China | 218,189 | - | 412 | - | 54.3 | 1990-1991 | China’s national Disease Surveillance Points | death | 15 | [3] |
| 5 | Shen, C. (2013) | Hong Kong | 22,680 | 44,140 | 311 | 279 | ≥65 | 1998-2001 | Death registration | death | 10.5 | [4] |
| 6 | Kono, S. (1987) | Japan | 5,130 | - | 51 | - | 49 | 1965 | Death certificates | death | 19 | [6] |
| 7 | Jee, S. H. (2004) | Korea | 823,158 | 459,954 | 3,807 | | 47.35 | 1992-1995 | National death certificate data | death | 10 | [23] |
| 8 | Allen, N. E. (2009) | UK | - | 1,280,296 | - | 337 | 55.9 | 1996-2001 | National Health Service (NHS) Central Registers | incidence | 7.2 | [9] |
| 9 | Yi, S. W. (2018) | Korea | 274,265 | 230,381 | 2,259 | 485 | 53 | 2002-2003 | Hospital discharge records from the NHIS | incidence | 10.5 | [24] |
| 10 | Klatsky, A. L. (2015) | USA | 55,040 | 69,153 | 213 | | 41 | 1978-1985 | KP Cancer Registry | incidence | 17.8 | [20] |
| *Pancreas cancer* | | | | | | | | | | | | |
| 1 | Yi, S. W. (2010) | Korea | 2,696 | 3,595 | 15 | 14 | 66.6 | 1985 | Death certificates | death | 20.8 | [1] |
| 2 | Kim, M. K. (2010) | Korea | 919,199 | 422,194 | 388 | 113 | 48.3 (M)/49 (F) | 2000 | National death certificate data | death | 5 | [2] |
| 3 | Heinen, M. M. (2009) | Netherlands | 58,279 | 62,573 | 185 | 165 | 55-69 | 1986 | Netherlands Cancer Registry and the Netherlands Pathology Registry | incidence | 13.3 | [25] |
| 4 | Shen, C. (2013) | Hong Kong | 22,680 | 44,140 | 94 | 148 | ≥65 | 1998-2001 | Death registration | death | 10.5 | [4] |
| 5 | Gapstur, S. M. (2011) | USA | 453,770 | 576,697 | 3,443 | 3,404 | ≥30 | 1982 | Death certificates | death | 24 | [26] |
| 6 | Nakamura, K. (2011) | Japan | 14,241 | 16,585 | 33 | 19 | 55.25 | 1992 | National Vital Statistics | death | 8 | [27] |
| 7 | Michaud, D. S._HPFS (2001) | USA | 47,794 | - | 130 | - | 40-75 | 1986 | Medical records or pathology reports | incidence | 12 | [28] |
| 8 | Michaud, D. S._NHS (2001) | USA | - | 88,799 | - | 158 | 30-55 | 1976 | Medical records or pathology reports | incidence | 16 | [28] |
| 9 | Stolzenberg-Solomon, R. Z. (2001) | Finland | 27,101 | - | 157 | - | 57.5 | 1985-1988 | Finnish Cancer Registry | incidence | 10.2 | [29] |
| 10 | Jayasekara, H. (2019) | Australia | 15,707 | 22,765 | 107 | 132 | 55.2 | 1990-1994 | Victorian Cancer Registry, Victorian Registry of Births, Deaths and Marriages, National Death Index and Australian Cancer Database | death/incidence | 20.2 | [30] |
| 11 | Gaziano, J. M. (2000) | USA | 89,299 | - | 78 | - | 40-84 | 1982-1983 | Death certificates | death | 5.46 | [31] |
| 12 | Allen, N. E. (2009) | UK | - | 1,280,296 | - | 1,325 | 55.9 | 1996-2001 | National Health Service (NHS) Central Registers | incidence | 7.2 | [9] |
| 13 | Kuzmickiene, I. (2013) | Lithuania | 7,132 | - | 77 | - | 52.6 | 1972-1974 | Lithuanian Cancer Registry and death certificates | incidence | 19.3 | [32] |
| 14 | Pang, Y. (2018) | China | 209,290 | 301,024 | 347 | 341 | 51.5 | 2004-2008 | China CDC's Disease Surveillance Points (DSP) system | death/incidence | 9 | [33] |
| 15 | Harnack, L. J. (1997) | USA | - | 33,976 | - | 66 | 61.7 | 1986 | National Cancer Institute’s Surveillance | incidence | 9 | [34] |
| 16 | Klatsky, A. L. (2015) | USA | 55,040 | 69,153 | 535 | | 41 | 1978-1985 | KP Cancer Registry | incidence | 17.8 | [20] |
| *Colorectal cancer* | | | | | | | | | | | | |
| 1 | Ma, E._cohort1 (2010) | Japan | 18,256 | - | 398 | - | 40-59 | 1995-2005 | Death certificates | death | 10.1 | [35] |
| 2 | Ma, E._cohort2 (2010) | Japan | 28,115 | - | 543 | - | 40-69 | 1993-2005 | Death certificates | death | 11 | [35] |
| 3 | Yi, S. W. (2010) | Korea | 2,696 | 3,595 | 26 | 16 | 66.6 | 1985 | Death certificates | death | 20.8 | [1] |
| 4 | Thun, M. J. (1997) | USA | 238,206 | 251,420 | 851 | 576 | 56 | 1982 | Death certificates | death | 9 | [36] |
| 5 | Kim, M. K. (2010) | Korea | 919,199 | - | 466 | - | 48.3 | 2000 | National death certificate data | death | 5 | [2] |
| 6 | Yang, L. (2012) | China | 218,189 | - | 63 | - | 54.3 | 1990-1991 | China’s national Disease Surveillance Points | death | 15 | [3] |
| 7 | Shen, C. (2013) | Hong Kong | 22,680 | 44,140 | 428 | 516 | ≥65 | 1998-2001 | Death registration | death | 10.5 | [4] |
| 8 | Tsong, W. H. (2007) | Singapore | 61,321 | | 845 | | 45-74 | 1993-1998 | Singapore Cancer Registry and Singapore Registry of Births and Deaths | death/incidence | 8.9 | [37] |
| 9 | Everatt, R. (2013) | Lithuania | 7,150 | - | 248 | - | 40-59 | 1972-1974, 1976-1980 | Lithuanian Cancer Registry and National and Regional Archives on Causes of Death | death/incidence | 30 | [38] |
| 10 | Kabat, G. C. (2008) | Canada | - | 49,654 | - | 617 | 40-59 | 1980-1985 | Canadian Cancer Database and the National Mortality Database | death/incidence | 16.4 | [39] |
| 11 | Breslow, R. A. (2011) | USA | 138,590 | 184,764 | 367 | 483 | ≥18  (for 1991, 18-44) | 1988 | National Center for Health Statistics | death | 18 | [40] |
| 12 | Nam, S. (2019) | Korea | 5,105,889 | 3,654,706 | 64,476 | 36,569 | - | 2004-2005 | Claims data from the NHIS | incidence | 8 | [41] |
| 13 | Toriola, A. T. (2008) | Finland | 2,682 | - | 59 | - | 53 | 1984-1989 | Finnish Cancer Registry | incidence | 16.7 | [42] |
| 14 | Akhter, M. (2007) | Japan | 21,199 | - | 307 | - | 40-64 | 1990 | Population registries of the 14 municipalities | incidence | 11 | [43] |
| 15 | Otani, T._cohort 1 (2003) | Japan | 27,063 | 27,435 | 244 | 259 | 40-59 | 1990 | Local major hospitals and population-based cancer registries | incidence | 9 | [44] |
| 16 | Otani, T._cohort 2 (2003) | Japan | 30,651 | 31,747 | 213 |  | 40-69 | 1993-1994 | Local major hospitals and population-based cancer registries | incidence | 6 | [44] |
| 17 | Bongaerts, B. W. (2008) | Netherlands | 58,279 | 62,573 | 2,323 | | 62.2 | 1986 | Netherlands Cancer Registry and to PALGA, a nationwide database of histo- and cytopathology reports | incidence | 13.3 | [45] |
| 18 | Ferrari, P. (2007) | Europe | 478,732 | | 1,833 | | 35-70 | 1992-1998 | Health insurance records, cancer and pathology registries | incidence | 6.2 | [46] |
| 19 | Thygesen, L. C. (2008) | USA | 47,432 | - | 868 | - | 40-75 | 1986 | Medical records | incidence | 16 | [47] |
| 20 | Cho, S. (2015) | Korea | 7,488 | 11,034 | 112 | 108 | ≥20 | 1993 | Korea Central Cancer Registry database and death certificate | death/incidence | 11.2 | [48] |
| 21 | Klatsky, A. L. (2015) | USA | 55,040 | 69,153 | 2,148 | | 41 | 1978-1985 | KP Cancer Registry | incidence | 17.8 | [20] |
| 22 | Park, J. Y. (2009) | Europe | 11,166 | 13,078 | 224 | 183 | 64 | 1993-1997 | Eastern Cancer Registration and Information Centre and the United Kingdom Office for National Statistics | incidence | 11 | [49] |
| 23 | Gaziano, J. M. (2000) | USA | 89,299 | - | 101 | - | 40-84 | 1982-1983 | Death certificates | death | 5.46 | [31] |
| 24 | Hippisley-Cox, J. (2015) | UK | 6,568,561 | | 18,617 | 13,570 | 25-84 | 1998 | National Statistics mortality records, National Statistics Cancer Registry | incidence | 15 | [50] |
| 25 | Sanjoaquin, M. A. (2004) | United Kingdom | 4,162 | 6,836 | 37 | 58 | 33 | 1980-1984 | UK National Health Service central register and cancer registration and death | incidence | 17 | [51] |
| 26 | Chen, K. (2005) | China | 31,087 | 33,256 | 121 | 121 | 61 | 1989-1990 | Cancer Registration System and a Rapid Reporting System from the CRC Registry | incidence | 10.6 | [52] |
| 27 | Razzak, A. A. (2011) | USA | 0 | 38,001 | 0 | 1,425 | 55-69 | 1986 | surveys and National Death Index | incidence | 12 | [53] |
| *Larynx cancer* | | | | | | | | | | | | |
| 1 | Kim, M. K. (2010) | Korea | 919,199 | - | 49 | - | 48.3 | 2000 | National death certificate data | death | 5 | [2] |
| 2 | Allen, N. E. (2009) | UK | - | 1,280,296 | - | 138 | 55.9 | 1996-2001 | National Health Service (NHS) Central Registers | incidence | 7.2 | [9] |
| *Lung cancer* | | | | | | | | | | | | |
| 1 | Nishino, Y. (2006) | Japan | 28,536 | - | 377 | - | 57.3 | 1988-1990 | Death certificates | death | 11 | [54] |
| 2 | Kim, M. K. (2010) | Korea | 919,199 | 422,194 | 1,700 | 222 | 48.3 (M)/ 49 (F) | 2000 | National death certificate data | death | 5 | [2] |
| 3 | Jung, E. J. (2012) | Korea | 6,405 | 9,915 | 123 | | ≥20 | 1993 | National death certificate data | death | 9.3 | [15] |
| 4 | Thun, M. J. (2009) | USA | 72,969 | 150,247 | 406 | 652 | ≥30 | 1982 | Death certificates | death | 24 | [55] |
| 5 | Yang, L. (2012) | China | 218,189 | - | 404 | - | 54.3 | 1990-1991 | China’s national Disease Surveillance Points | death | 15 | [3] |
| 6 | Shen, C. (2013) | Hong Kong | 22,680 | 44,140 | 762 | 1,022 | ≥65 | 1998-2001 | Death registration | death | 10.5 | [4] |
| 7 | Prescott, E. (1999) | Denmark | 15,107 | 13,053 | 480 | 194 | ≥20 | 1976-1978 | Danish Cancer Register | incidence | 29 | [56] |
| 8 | Kono, S. (1987) | Japan | 5,130 | - | 74 | - | 49 | 1965 | Death certificates | death | 19 | [6] |
| 9 | Kabat, G. C. (2008) | Canada | - | 49,654 | - | 358 | 40-59 | 1980-1985 | Canadian Cancer Database and the National Mortality Database | death/incidence | 16.4 | [39] |
| 10 | Rohrmann, S. (2006) | Europe | 142,798 | 335,792 | 606 | 513 | - | 1992-2000 | Health insurance records, and cancer and pathology registries or cancer registries or mortality registries | incidence | 6.4 | [57] |
| 11 | Gaziano, J. M. (2000) | USA | 89,299 | - | 181 | - | 40-84 | 1982-1983 | Death certificates | death | 5.46 | [31] |
| 12 | Allen, N. E. (2009) | UK | - | 1,280,296 | - | 5,203 | 55.9 | 1996-2001 | National Health Service (NHS) Central Registers | incidence | 7.2 | [9] |
| 13 | Breslow, R. A. (2011) | USA | 138,590 | 184,764 | 1,299 | 1,101 | ≥18  (for 1991, 18-44) | 1988 | National Center for Health Statistics | death | 18 | [40] |
| 14 | Viner, B. (2019) | Canada | 10,026 | 15,581 | 70 | 129 | 35-69 | 2000-2008 | Alberta Cancer Registry | incidence | 12.3 | [58] |
| 15 | Stemmermann, G. N. (1990) | USA | 8,006 | - | 209 | - | - | 1965-1968 | Hospital records, death certificates, and the Hawaii Tumor Registry, a statewide cancer registry | incidence | 21(~1989) | [18] |
| 16 | Djousse, L._original (2002) | USA | 4,265 | | 194 | | 41.6  (case) | 1948 | Self-reports and the National Death Index | incidence | 32.8 | [59] |
| 17 | Djousse, L._offspring (2002) | USA | 4,973 | | 75 | | 60.6  (case) | 1971 | Self-reports and the National Death Index | incidence | 16.2 | [59] |
| 18 | Klatsky, A. L. (2015) | USA | 55,040 | 69,153 | 1,989 | | 41 | 1978-1985 | KP Cancer Registry | incidence | 17.8 | [20] |
| 19 | Hippisley-Cox, J. (2015) | UK | 6,568,561 | | 18,617 | 13,570 | 25-84 | 1998 | National Statistics mortality records, National Statistics Cancer Registry | incidence | 15 | [50] |
| 20 | Troche, J. R. (2016) | USA | 294,246 | 198,656 | 10,227 | | 62 | 1995-1996 | State cancer registries | incidence | 10 | [60] |
| *Thyroid cancer* | | | | | | | | | | | | |
| 1 | Allen, N. E. (2009) | UK | - | 1,280,296 | - | 421 | 55.9 | 1996-2001 | National Health Service (NHS) Central Registers | incidence | 7.2 | [9] |
| 2 | Navarro Silvera, S. A. (2005) | Canada | - | 89,797 | - | 169 | 48.25 | 1980-1985 | Linkages to national cancer and mortality databases | death/incidence | 15.9 | [61] |
| 3 | Kabat, G. C. (2012) | Canada | - | 159,340 | - | 331 | 62.55 | 1993-1998 | Self-reports, medical records and pathology reports | incidence | 12.7 | [62] |
| 4 | Meinhold, C. L. (2009) | USA | 292,101 | 198,058 | 170 | 200 | 50-71 | 1995-1996 | Self-reports and the MyPyramid Servings database | incidence | 7.5 | [63] |
| 5 | Klatsky, A. L. (2015) | USA | 55,040 | 69,153 | 172 | | 41 | 1978-1985 | KP Cancer Registry | incidence | 17.8 | [20] |
| *Prostate cancer* | | | | | | | | | | | | |
| 1 | Schuurman, A. G. (1999) | Netherlands | 58,279 | - | 680 | - | 55-69 | 1986 | Dutch national database of pathology reports | incidence | 6.3 | [64] |
| 2 | Breslow, R. A._cohort 1 (1999) | USA | 5,766 | - | 252 | - | 25-74 | 1971-1975 | National Death Index | incidence | 17.1 | [65] |
| 3 | Breslow, R. A._cohort 2 (1999) | USA | 3,775 | - | 134 | - | 25-74 | 1982-1984 | National Death Index | incidence | 9 | [65] |
| 4 | Sawada, N. (2014) | Japan | 48,218 | - | 913 | - | 40-69 | 1990, 1993 | Cancer registries | incidence | 16 | [66] |
| 5 | Kim, M. K. (2010) | Korea | 919,199 | - | 46 | - | 48.3 | 2000 | National death certificate data | death | 5 | [2] |
| 6 | Baglietto, L. (2006) | Australia | 16,872 | - | 785 | - | 27-70 | 1990-1994 | Victorian Cancer Registry or National Death Index | death/incidence | 10.3 | [67] |
| 7 | Sesso, H. D. (2001) | USA | 7,612 | - | 366 | - | 66.6 | 1988 | Self-reports or physician-diagnosed | incidence | 5 | [68] |
| 8 | Rohrmann, S. (2008) | Europe | 142,607 | - | 2,655 | - | 40-65 | 1992-2000 | Cancer or mortality registries | death/incidence | 8.7 | [69] |
| 9 | Hiatt, R. A. (1994) | USA | 43,432 | - | 238 | - | 47.4 | 1979-1985 | California Tumor Registry | incidence | 4.6 | [70] |
| 10 | Shen, C. (2013) | Hong Kong | 22,680 | 44,140 | 181 | - | ≥65 | 1998-2001 | Death registration | death | 10.5 | [4] |
| 11 | Platz, E. A. (2004) | USA | 47,843 | - | 2,479 | - | 40-75 | 1986 | National Death Index | death | 13 | [71] |
| 12 | Velicer, C. M. (2006) | USA | 34,565 | - | 816 | - | 61.7 | 2000-2002 | Cancer registry | incidence | 4 | [72] |
| 13 | Albertsen, K. & Gronbaek, M. (2002) | Denmark | 12,989 | - | 233 | - | 52 | 1976 | Danish Cancer Registry | incidence | 12.3 | [73] |
| 14 | Gaziano, J. M. (2000) | USA | 89,299 | - | 74 | - | 40-84 | 1982-1983 | Death certificates | death | 5.46 | [31] |
| 15 | Breslow, R. A. (2011) | USA | 138,590 | 184,764 | 438 | - | ≥18  (for 1991, 18-44) | 1988 | National Center for Health Statistics | death | 18 | [40] |
| 16 | Viner, B. (2019) | Canada | 10,026 | 15,581 | 393 | - | 35-69 | 2000-2008 | Alberta Cancer Registry | incidence | 12.3 | [58] |
| 17 | Stemmermann, G. N. (1990) | USA | 8,006 | - | 227 | - | - | 1940-1942 | hospital records, death certificates, and the Hawaii Tumor Registry, a statewide cancer registry | incidence | 46 (~1988) | [18] |
| 18 | Gong, Z. (2009) | USA | 10,920 | - | 2,129 | - | ≥55 | - | Self-reports | incidence | 7 | [74] |
| 19 | Klatsky, A. L. (2015) | USA | 55,040 | 69,153 | 3,408 | - | 41 | 1978-1985 | KP Cancer Registry | incidence | 17.8 | [20] |
| 20 | Watters, J. L. (2010) | USA | 294,707 | - | 17,227 | - | 50-71 | 1995-1996 | State cancer registry databases | incidence | 7 | [75] |
| 21 | Weinstein, S. J. (2006) | Finland | 27,111 | - | 1,270 | - | 50-69 | 1985 | Finnish Cancer Registry | incidence | ≤17 | [76] |
| *Breast cancer* | | | | | | | | | | | | |
| 1 | Jain, M. G. (2000) | Canada | - | 49,165 | - | 223 | 40-59 | 1980-1985 | National Mortality Database | death | 10.3 | [77] |
| 2 | van den Brandt, P. (1995) | Netherlands | - | 62,573 | - | 422 | 55-69 | 1986 | Cancer registries and a pathology register | incidence | 3.3 | [78] |
| 3 | Zhang, S. M. (2007) | USA | - | 38,454 | - | 1,484 | ≥45 | 1992 | National Death Index and medical records | incidence | 10 | [79] |
| 4 | Kim, H. J. (2017) | USA | - | 93,835 | - | 2,866 | 25-42 | 1989 | National Death Index | incidence | 20 | [80] |
| 5 | Thun, M. J. (1997) | USA | 238,206 | 251,420 | - | 691 | 56 | 1982 | Death certificates | death | 9 | [36] |
| 6 | Fuchs, C. S. (1995) | USA | - | 85,709 | - | 2,658 | 34-59 | 1980 | National Death Index and death certificates | death | 12 | [81] |
| 7 | Kim, M. K. (2010) | Korea | - | 422,194 | - | 72 | 49 | 2000 | National death certificate data | death | 5 | [2] |
| 8 | Kabat, G. C. (2008) | Canada | - | 49,654 | - | 2,491 | 40-59 | 1980-1985 | Canadian Cancer Database and the National Mortality Database | death/incidence | 16.4 | [39] |
| 9 | Sinnadurai, S. (2020) | Japan | - | 33,396 | - | 245 | 57.7 | 1988-1990 | Cancer registries | incidence | 20 | [82] |
| 10 | Heberg, J. (2019) | Denmark | - | 16,106 | - | 1,407 | 56 | 1993 | Hospital diagnosis records | incidence | 18.8 | [83] |
| 11 | Chen, W. Y. (2011) | USA | - | 74,854 | - | 7,690 | 30-55 | 1976 | Pathology reports and National Death Index | incidence | 28.5 | [84] |
| 12 | Allen, N. E. (2009) | UK | - | 1,280,296 | - | 28,380 | 55.9 | 1996-2001 | National Health Service (NHS) Central Registers | incidence | 7.2 | [9] |
| 13 | Breslow, R. A. (2011) | USA | 138,590 | 184,764 | - | 677 | ≥18  (for 1991, 18-44) | 1988 | National Center for Health Statistics | death | 18 | [40] |
| 14 | Suzuki, R. (2005) | Sweden | - | 51,847 | - | 1,188 | - | 1987-1989 | National and Regional Cancer Registries | incidence | 8.3 | [85] |
| 15 | Lew, J. Q. (2009) | USA | - | 184,418 | - | 5,461 | 50-71 | 1995-1996 | Cancer registries | incidence | 7 | [86] |
| 16 | Rohan, T. E. (2000) | Canada | - | 56,837 | - | 1,469 | 40-59 | 1980-1985 | Cancer registry | incidence | 10 | [87] |
| 17 | Li, C. I. (2010) | USA | - | 87,724 | - | 2,944 | 50-79 | 1993-1998 | medical records | incidence | 7~12 | [88] |
| 18 | Simon, M. S. (1991) | USA | - | 1,954 | - | 87 | ≥21 | 1959-1960 | Self-report | incidence | 28 | [89] |
| 19 | Garland, M. (1999) | USA | - | 116,671 | - | 445 | 25-42 | 1989 | Pathology reports | incidence | 6 | [90] |
| 20 | Feigelson, H. S. (2001) | USA | - | 242,010 | - | 1,442 | - | 1982 | National Death Index and death certificates | death/incidence | 14 | [91] |
| 21 | Suzuki, R. (2010) | Japan | - | 50,757 | - | 572 | 40–69 | 1990-1994 | Self-report | incidence | 15 | [92] |
| 22 | Morch, L. S. (2007) | Denmark | - | 134,796 | - | 457 | ≥44 | 1968 | Nationwide Danish registers | incidence | 7.6 | [93] |
| 23 | Petri, A. L. (2004) | Denmark | - | 13,074 | - | 473 | 20-91 | 1976-1978 | Danish Cancer Registry | incidence | 6.1 | [94] |
| 24 | Feigelson, H. S. (2003) | USA | - | 66,561 | - | 1,303 | 62.6 | 1992 | National Death Index and death certificates | death/incidence | 5 | [95] |
| 25 | Baglietto, L. (2005) | Australia | - | 17,447 | - | 537 | 54.7 | 1990-1994 | Victorian cancer registry | incidence | 10.1 | [96] |
| 26 | Horn-Ross, P. L. (2002) | USA | - | 111,526 | - | 711 | 52.5 | 1995-1996 | California Cancer Registry | incidence | 1.99 | [97] |
| 27 | Hoyer, A. P. (1992) | Denmark | - | 5,207 | - | 51 | 30-80 | 1982 | Danish Cancer Registry | incidence | 26 | [98] |
| 28 | Li, Y. (2009) | USA | - | 70,033 | - | 2,829 | 40.6 | 1978-1985 | Health Care Progrmme's Cancer Registry | death/incidence | 16 | [99] |
| 29 | Thygesen, L. C. (2008) | Denmark | - | 9,318 | - | 476 | - | 1976 | National Central Person Registry and the Danish Cancer Registry | incidence | 27 | [100] |
| 30 | Falk, R. T. (2014) | USA | - | 54,562 | - | 2,372 | 55-74 | 1993-2001 | National Death Index and death certificates | incidence | 8.9 | [101] |
| 31 | Park, S. Y. (2014) | USA | - | 85,089 | - | 3,885 | 45-75 | 1993-1996 | Surveillance, Epidemiology, and End Results | incidence | 12.4 | [102] |
| 32 | Fagherazzi, G. (2015) | France | - | 66,481 | - | 2,812 | 52.7 | 1990-1991 | Self-report | incidence | 15 | [103] |
| 33 | Shin, A. (2015) | Sweden | - | 45,233 | - | 1,385 | 30-49 | 1991-1992 | Nationwide health registries in Sweden | incidence | 13 | [104] |
| 34 | Klatsky, A. L. (2015) | USA | 55,040 | 69,153 | - | 3,639 | 41 | 1978-1985 | KP Cancer Registry | incidence | 17.8 | [20] |
| 35 | Hippisley-Cox, J. (2015) | UK | 2,447,866 | 2,495,899 | - | 41,315 | 25–84 | 1998 | National Statistics mortality records, National Statistics Cancer Registry | death/incidence | 15 | [50] |
| 36 | Chhim, A. S. (2015) | France | - | 3,771 | - | 158 | 35-60 | 1994-1995 | Pathology reports | incidence | 12.1 | [105] |

Reference list

1. Yi, S. W., Sull, J. W., Linton, J. A., Nam, C. M., & Ohrr, H. (2010). Alcohol consumption and digestive cancer mortality in Koreans: the Kangwha Cohort Study. *Journal of epidemiology*, *20*(3), 204-211.
2. Kim, M. K., Ko, M. J., & Han, J. T. (2010). Alcohol consumption and mortality from all-cause and cancers among 1.34 million Koreans: the results from the Korea national health insurance corporation’s health examinee cohort in 2000. *Cancer Causes & Control*, *21*, 2295-2302.
3. Yang, L., Zhou, M., Sherliker, P., Cai, Y., Peto, R., Wang, L., ... & Chen, Z. (2012). Alcohol drinking and overall and cause-specific mortality in China: nationally representative prospective study of 220 000 men with 15 years of follow-up. *International journal of epidemiology*, *41*(4), 1101-1113.
4. Shen, C., Schooling, C. M., Chan, W. M., Xu, L., Lee, S. Y., & Lam, T. H. (2013). Alcohol intake and death from cancer in a prospective Chinese elderly cohort study in Hong Kong. *J Epidemiol Community Health*, *67*(10), 813-820.
5. Fan, Y., Yuan, J. M., Wang, R., Gao, Y. T., & Yu, M. C. (2008). Alcohol, tobacco, and diet in relation to esophageal cancer: the Shanghai Cohort Study. *Nutrition and cancer*, *60*(3), 354-363.
6. Kono, S., Ikeda, M., Tokudome, S., Nishizumi, M., & Kuratsune, M. (1987). Cigarette smoking, alcohol and cancer mortality: a cohort study of male Japanese physicians. *Japanese Journal of Cancer Research GANN*, *78*(12), 1323-1328.
7. Yi, S. W., Hong, J. S., Yi, J. J., & Ohrr, H. (2016). Impact of alcohol consumption and body mass index on mortality from nonneoplastic liver diseases, upper aerodigestive tract cancers, and alcohol use disorders in Korean older middle-aged men: prospective cohort study. *Medicine*, *95*(39).
8. Yaegashi, Y., Onoda, T., Morioka, S., Hashimoto, T., Takeshita, T., Sakata, K., & Tamakoshi, A. (2014). Joint effects of smoking and alcohol drinking on esophageal cancer mortality in Japanese men: findings from the Japan collaborative cohort study. *Asian Pacific Journal of Cancer Prevention*, *15*(2), 1023-1029.
9. Allen, N. E., Beral, V., Casabonne, D., Kan, S. W., Reeves, G. K., Brown, A., & Green, J. (2009). Moderate alcohol intake and cancer incidence in women. *Journal of the National Cancer Institute*, *101*(5), 296-305.
10. Kinjo, Y., Cui, Y., Akiba, S., Watanabe, S., Yamaguchi, N., Sobue, T., ... & Beral, V. (1998). Mortality risks of oesophageal cancer associated with hot tea, alcohol, tobacco and diet in Japan. *Journal of epidemiology*, *8*(4), 235-243.
11. Sakata, K., Hoshiyama, Y., Morioka, S., Hashimoto, T., Takeshita, T., & Tamakoshi, A. (2005). Smoking, alcohol drinking and esophageal cancer: findings from the JACC Study. *Journal of epidemiology*, *15*(Supplement_II), S212-S219.
12. Kimm, H., Kim, S., & Jee, S. H. (2010). The independent effects of cigarette smoking, alcohol consumption, and serum aspartate aminotransferase on the alanine aminotransferase ratio in korean men for the risk for esophageal cancer. *Yonsei Medical Journal*, *51*(3), 310-317.
13. Boffetta, P., & Garfinkel, L. (1990). Alcohol drinking and mortality among men enrolled in an American Cancer Society prospective study. *Epidemiology*, 342-348.
14. Ishiguro, S., Sasazuki, S., Inoue, M., Kurahashi, N., Iwasaki, M., Tsugane, S., & JPHC Study Group. (2009). Effect of alcohol consumption, cigarette smoking and flushing response on esophageal cancer risk: a population-based cohort study (JPHC study). *Cancer letters*, *275*(2), 240-246.
15. Jung, E. J., Shin, A., Park, S. K., Ma, S. H., Cho, I. S., Park, B., ... & Yoo, K. Y. (2012). Alcohol consumption and mortality in the Korean multi-center cancer cohort study. *Journal of Preventive Medicine and Public Health*, *45*(5), 301.
16. Ma, S. H., Jung, W., Weiderpass, E., Jang, J., Hwang, Y., Ahn, C., ... & Park, S. K. (2015). Impact of alcohol drinking on gastric cancer development according to Helicobacter pylori infection status. *British journal of cancer*, *113*(9), 1381-1388.
17. Sung, N. Y., Choi, K. S., Park, E. C., Park, K., Lee, S. Y., Lee, A. K., ... & Shin, H. R. (2007). Smoking, alcohol and gastric cancer risk in Korean men: the National Health Insurance Corporation Study. *British journal of cancer*, *97*(5), 700-704.
18. Stemmermann, G. N., Nomura, A. M., Chyou, P. H., & Yoshizawa, C. (1990). Prospective study of alcohol intake and large bowel cancer. *Digestive diseases and sciences*, *35*(11), 1414-1420.
19. Moy, K. A., Fan, Y., Wang, R., Gao, Y. T., Yu, M. C., & Yuan, J. M. (2010). Alcohol and tobacco use in relation to gastric cancer: a prospective study of men in Shanghai, China. *Cancer epidemiology, biomarkers & prevention*, *19*(9), 2287-2297.
20. Klatsky, A. L., Li, Y., Tran, H. N., Baer, D., Udaltsova, N., Armstrong, M. A., & Friedman, G. D. (2015). Alcohol intake, beverage choice, and cancer: a cohort study in a large kaiser permanente population. *The Permanente Journal*, *19*(2), 28.
21. Nomura, A. M., Stemmermann, G. N., & Chyou, P. H. (1995). Gastric cancer among the Japanese in Hawaii. *Japanese journal of cancer research*, *86*(10), 916-923.
22. Galanis, D. J., Kolonel, L. N., Lee, J., & Nomura, A. (1998). Intakes of selected foods and beverages and the incidence of gastric cancer among the Japanese residents of Hawaii: a prospective study. *International journal of epidemiology*, *27*(2), 173-180.
23. Jee, S. H., Ohrr, H., Sull, J. W., & Samet, J. M. (2004). Cigarette smoking, alcohol drinking, hepatitis B, and risk for hepatocellular carcinoma in Korea. *Journal of the national cancer institute*, *96*(24), 1851-1856.
24. Yi, S. W., Choi, J. S., Yi, J. J., Lee, Y. H., & Han, K. J. (2018). Risk factors for hepatocellular carcinoma by age, sex, and liver disorder status: a prospective cohort study in Korea. *Cancer*, *124*(13), 2748-2757.
25. Heinen, M. M., Verhage, B. A., Ambergen, T. A., Goldbohm, R. A., & van den Brandt, P. A. (2009). Alcohol consumption and risk of pancreatic cancer in the Netherlands cohort study. *American journal of epidemiology*, *169*(10), 1233-1242.
26. Gapstur, S. M., Jacobs, E. J., Deka, A., McCullough, M. L., Patel, A. V., & Thun, M. J. (2011). Association of alcohol intake with pancreatic cancer mortality in never smokers. *Archives of internal medicine*, *171*(5), 444-451.
27. Nakamura, K., Nagata, C., Wada, K., Tamai, Y., Tsuji, M., Takatsuka, N., & Shimizu, H. (2011). Cigarette smoking and other lifestyle factors in relation to the risk of pancreatic cancer death: a prospective cohort study in Japan. *Japanese journal of clinical oncology*, *41*(2), 225-231.
28. Michaud, D. S., Giovannucci, E., Willett, W. C., Colditz, G. A., & Fuchs, C. S. (2001). Coffee and alcohol consumption and the risk of pancreatic cancer in two prospective United States cohorts. *Cancer Epidemiology Biomarkers & Prevention*, *10*(5), 429-437.
29. Stolzenberg-Solomon, R. Z., Pietinen, P., Barrett, M. J., Taylor, P. R., Virtamo, J., & Albanes, D. (2001). Dietary and other methyl-group availability factors and pancreatic cancer risk in a cohort of male smokers. *American journal of epidemiology*, *153*(7), 680-687.
30. Jayasekara, H., English, D. R., Hodge, A. M., Room, R., Hopper, J. L., Milne, R. L., ... & MacInnis, R. J. (2019). Lifetime alcohol intake and pancreatic cancer incidence and survival: findings from the Melbourne Collaborative Cohort Study. *Cancer Causes & Control*, *30*, 323-331.
31. Gaziano, J. M., Gaziano, T. A., Glynn, R. J., Sesso, H. D., Ajani, U. A., Stampfer, M. J., ... & Hennekens, C. H. (2000). Light-to-moderate alcohol consumption and mortality in the Physicians’ Health Study enrollment cohort. *Journal of the American College of Cardiology*, *35*(1), 96-105.
32. Kuzmickiene, I., Everatt, R., Virviciute, D., Tamosiunas, A., Radisauskas, R., Reklaitiene, R., & Milinaviciene, E. (2013). Smoking and other risk factors for pancreatic cancer: a cohort study in men in Lithuania. *Cancer epidemiology*, *37*(2), 133-139.
33. Pang, Y., Holmes, M. V., Guo, Y., Yang, L., Bian, Z., Chen, Y., ... & Chen, Z. (2018). Smoking, alcohol, and diet in relation to risk of pancreatic cancer in China: a prospective study of 0.5 million people. *Cancer medicine*, *7*(1), 229-239.
34. Harnack, L. J., Anderson, K. E., Zheng, W., Folsom, A. R., Sellers, T. A., & Kushi, L. H. (1997). Smoking, alcohol, coffee, and tea intake and incidence of cancer of the exocrine pancreas: the Iowa Women's Health Study. *Cancer epidemiology, biomarkers & prevention: a publication of the American Association for Cancer Research, cosponsored by the American Society of Preventive Oncology*, *6*(12), 1081-1086.
35. Ma, E., Sasazuki, S., Iwasaki, M., Sawada, N., Inoue, M., & Shoichiro Tsugane for the Japan Public Health Center-based Prospective Study Group. (2010). 10-Year risk of colorectal cancer: development and validation of a prediction model in middle-aged Japanese men. *Cancer epidemiology*, *34*(5), 534-541.
36. Thun, M. J., Peto, R., Lopez, A. D., Monaco, J. H., Henley, S. J., Heath Jr, C. W., & Doll, R. (1997). Alcohol consumption and mortality among middle-aged and elderly US adults. *New England Journal of Medicine*, *337*(24), 1705-1714.
37. Tsong, W. H., Koh, W. P., Yuan, J. M., Wang, R., Sun, C. L., & Yu, M. C. (2007). Cigarettes and alcohol in relation to colorectal cancer: the Singapore Chinese Health Study. *British journal of cancer*, *96*(5), 821-827.
38. Everatt, R., Tamosiunas, A., Virviciute, D., Kuzmickiene, I., & Reklaitiene, R. (2013). Consumption of alcohol and risk of cancer among men: a 30 year cohort study in Lithuania. *European journal of epidemiology*, *28*, 383-392.
39. Kabat, G. C., Miller, A. B., Jain, M., & Rohan, T. E. (2008). Dietary intake of selected B vitamins in relation to risk of major cancers in women. *British journal of cancer*, *99*(5), 816-821.
40. Breslow, R. A., Chen, C. M., Graubard, B. I., & Mukamal, K. J. (2011). Prospective study of alcohol consumption quantity and frequency and cancer-specific mortality in the US population. *American journal of epidemiology*, *174*(9), 1044-1053.
41. Nam, S., Choi, Y. J., Kim, D. W., Park, E. C., & Kang, J. G. (2019). Risk factors for colorectal cancer in Korea: a population-based retrospective cohort study. *Annals of coloproctology*, *35*(6), 347.
42. Toriola, A. T., Kurl, S., Laukanen, J. A., Mazengo, C., & Kauhanen, J. (2008). Alcohol consumption and risk of colorectal cancer: the Findrink study. *European journal of epidemiology*, *23*, 395-401.
43. Akhter, M., Kuriyama, S., Nakaya, N., Shimazu, T., Ohmori, K., Nishino, Y., ... & Tsuji, I. (2007). Alcohol consumption is associated with an increased risk of distal colon and rectal cancer in Japanese men: the Miyagi Cohort Study. *European Journal of Cancer*, *43*(2), 383-390.
44. Otani, T., Iwasaki, M., Yamamoto, S., Sobue, T., Hanaoka, T., Inoue, M., ... & Japan Public Health Center-based Prospective Study Group. (2003). Alcohol consumption, smoking, and subsequent risk of colorectal cancer in middle-aged and elderly Japanese men and women: Japan Public Health Center-based prospective study. *Cancer Epidemiology Biomarkers & Prevention*, *12*(12), 1492-1500.
45. Bongaerts, B. W., van den Brandt, P. A., Goldbohm, R. A., de Goeij, A. F., & Weijenberg, M. P. (2008). Alcohol consumption, type of alcoholic beverage and risk of colorectal cancer at specific subsites. *International Journal of Cancer*, *123*(10), 2411-2417.
46. Ferrari, P., Jenab, M., Norat, T., Moskal, A., Slimani, N., Olsen, A., ... & Riboli, E. (2007). Lifetime and baseline alcohol intake and risk of colon and rectal cancers in the European prospective investigation into cancer and nutrition (EPIC). *International journal of cancer*, *121*(9), 2065-2072.
47. Thygesen, L. C., Wu, K., Grønbœk, M., Fuchs, C. S., Willett, W. C., & Giovannucci, E. (2008). Alcohol intake and colorectal cancer: a comparison of approaches for including repeated measures of alcohol consumption. *Epidemiology*, 258-264.
48. Cho, S., Shin, A., Park, S. K., Shin, H. R., Chang, S. H., & Yoo, K. Y. (2015). Alcohol drinking, cigarette smoking and risk of colorectal cancer in the Korean multi-center cancer cohort. *Journal of cancer prevention*, *20*(2), 147.
49. Park, J. Y., Mitrou, P. N., Dahm, C. C., Luben, R. N., Wareham, N. J., Khaw, K. T., & Rodwell, S. A. (2009). Baseline alcohol consumption, type of alcoholic beverage and risk of colorectal cancer in the European Prospective Investigation into Cancer and Nutrition-Norfolk study. *Cancer epidemiology*, *33*(5), 347-354.
50. Hippisley-Cox, J., & Coupland, C. (2015). Development and validation of risk prediction algorithms to estimate future risk of common cancers in men and women: prospective cohort study. *BMJ open*, *5*(3), e007825.
51. Sanjoaquin, M. A., Appleby, P. N., Thorogood, M., Mann, J. I., & Key, T. J. (2004). Nutrition, lifestyle and colorectal cancer incidence: a prospective investigation of 10 998 vegetarians and non-vegetarians in the United Kingdom. British journal of cancer, 90(1), 118-121.
52. Chen, K., Jiang, Q., Ma, X., Li, Q., Yao, K., Yu, W., & Zheng, S. (2005). Alcohol drinking and colorectal cancer: a population-based prospective cohort study in China. European journal of epidemiology, 20, 149-154.
53. Razzak, A. A., Oxentenko, A. S., Vierkant, R. A., Tillmans, L. S., Wang, A. H., Weisenberger, D. J., ... & Limburg, P. J. (2011). Alcohol intake and colorectal cancer risk by molecularly defined subtypes in a prospective study of older women. Cancer Prevention Research, 4(12), 2035-2043.
54. Nishino, Y., Wakai, K., Kondo, T., Seki, N., Yoshinori, I., Suzuki, K., ... & Tamakoshi, A. (2006). Alcohol consumption and lung cancer mortality in Japanese men: results from Japan collaborative cohort (JACC) study. *Journal of epidemiology*, *16*(2), 49-56.
55. Thun, M. J., Hannan, L. M., & DeLancey, J. O. L. (2009). Alcohol consumption not associated with lung cancer mortality in lifelong nonsmokers. *Cancer epidemiology, biomarkers & prevention*, *18*(8), 2269-2272.
56. Prescott, E., Grønbæk, M., Becker, U., & Sørensen, T. I. (1999). Alcohol intake and the risk of lung cancer: influence of type of alcoholic beverage. *American journal of epidemiology*, *149*(5), 463-470.
57. Rohrmann, S., Linseisen, J., Boshuizen, H. C., Whittaker, J., Agudo, A., Vineis, P., ... & Riboli, E. (2006). Ethanol intake and risk of lung cancer in the European Prospective Investigation into Cancer and Nutrition (EPIC). *American journal of epidemiology*, *164*(11), 1103-1114.
58. Viner, B., Barberio, A. M., Haig, T. R., Friedenreich, C. M., & Brenner, D. R. (2019). The individual and combined effects of alcohol consumption and cigarette smoking on site-specific cancer risk in a prospective cohort of 26,607 adults: results from Alberta’s Tomorrow Project. *Cancer Causes & Control*, *30*, 1313-1326.
59. Djoussé, L., Dorgan, J. F., Zhang, Y., Schatzkin, A., Hood, M., D’Agostino, R. B., ... & Ellison, R. C. (2002). Alcohol consumption and risk of lung cancer: the Framingham Study. *Journal of the National Cancer Institute*, *94*(24), 1877-1882.
60. Troche, J. R., Mayne, S. T., Freedman, N. D., Shebl, F. M., & Abnet, C. C. (2016). The association between alcohol consumption and lung carcinoma by histological subtype. *American journal of epidemiology*, *183*(2), 110-121.
61. Navarro Silvera, S. A., Miller, A. B., & Rohan, T. E. (2005). Risk factors for thyroid cancer: a prospective cohort study. *International journal of cancer*, *116*(3), 433-438.
62. Kabat, G. C., Kim, M. Y., Wactawski-Wende, J., & Rohan, T. E. (2012). Smoking and alcohol consumption in relation to risk of thyroid cancer in postmenopausal women. *Cancer epidemiology*, *36*(4), 335-340.
63. Meinhold, C. L., Park, Y., Stolzenberg-Solomon, R. Z., Hollenbeck, A. R., Schatzkin, A., & Berrington de Gonzalez, A. (2009). Alcohol intake and risk of thyroid cancer in the NIH-AARP Diet and Health Study. *British journal of cancer*, *101*(9), 1630-1634.
64. Schuurman, A. G., Goldbohm, R. A., & van den Brandt, P. A. (1999). A prospective cohort study on consumption of alcoholic beverages in relation to prostate cancer incidence (The Netherlands). *Cancer Causes & Control*, *10*, 597-605.
65. Breslow, R. A., Wideroff, L., Graubard, B. I., Erwin, D., Reichman, M. E., Ziegler, R. G., & Ballard-Barbash, R. (1999). Alcohol and prostate cancer in the NHANES I epidemiologic follow-up study. *Annals of epidemiology*, *9*(4), 254-261.
66. Sawada, N., Inoue, M., Iwasaki, M., Sasazuki, S., Yamaji, T., Shimazu, T., & Tsugane, S. (2014). Alcohol and smoking and subsequent risk of prostate cancer in Japanese men: the Japan Public Health Center‐based prospective study. *International journal of cancer*, *134*(4), 971-978.
67. Baglietto, L., Severi, G., English, D. R., Hopper, J. L., & Giles, G. G. (2006). Alcohol consumption and prostate cancer risk: results from the Melbourne collaborative cohort study. *International journal of cancer*, *119*(6), 1501-1504.
68. Sesso, H. D., Paffenbarger Jr, R. S., & Lee, I. M. (2001). Alcohol consumption and risk of prostate cancer: The Harvard Alumni Health Study. *International journal of epidemiology*, *30*(4), 749-755.
69. Rohrmann, S., Linseisen, J., Key, T. J., Jensen, M. K., Overvad, K., Johnsen, N. F., ... & Riboli, E. (2008). Alcohol consumption and the risk for prostate cancer in the European Prospective Investigation into Cancer and Nutrition. *Cancer Epidemiology Biomarkers & Prevention*, *17*(5), 1282-1287.
70. Hiatt, R. A., Anne Armstrong, M., Klatsky, A. L., & Sidney, S. (1994). Alcohol consumption, smoking, and other risk factors and prostate cancer in a large health plan cohort in California (United States). *Cancer Causes & Control*, *5*, 66-72.
71. Platz, E. A., Leitzmann, M. F., Rimm, E. B., Willett, W. C., & Giovannucci, E. (2004). Alcohol intake, drinking patterns, and risk of prostate cancer in a large prospective cohort study. *American journal of epidemiology*, *159*(5), 444-453.
72. Velicer, C. M., Kristal, A., & White, E. (2006). Alcohol use and the risk of prostate cancer: results from the VITAL cohort study. *Nutrition and cancer*, *56*(1), 50-56.
73. Albertsen, K., & Grønbæk, M. (2002). Does amount or type of alcohol influence the risk of prostate cancer?. *The prostate*, *52*(4), 297-304.
74. Gong, Z., Kristal, A. R., Schenk, J. M., Tangen, C. M., Goodman, P. J., & Thompson, I. M. (2009). Alcohol consumption, finasteride, and prostate cancer risk: results from the Prostate Cancer Prevention Trial. *Cancer: Interdisciplinary International Journal of the American Cancer Society*, *115*(16), 3661-3669.
75. Watters, J. L., Park, Y., Hollenbeck, A., Schatzkin, A., & Albanes, D. (2010). Alcoholic beverages and prostate cancer in a prospective US cohort study. *American journal of epidemiology*, *172*(7), 773-780.
76. Weinstein, S. J., Stolzenberg-Solomon, R., Pietinen, P., Taylor, P. R., Virtamo, J., & Albanes, D. (2006). Dietary factors of one-carbon metabolism and prostate cancer risk. *The American journal of clinical nutrition*, *84*(4), 929-935.
77. Jain, M. G., Ferrence, R. G., Rehm, J. T., Bondy, S. J., Rohan, T. E., Ashley, M. J., ... & Miller, A. B. (2000). Alcohol and breast cancer mortality in a cohort study. *Breast cancer research and treatment*, *64*, 201-209.
78. van den Brandt, P., Goldbohm, R. A., & van't Veer, P. (1995). Alcohol and breast cancer: results from The Netherlands Cohort Study. *American Journal of Epidemiology*, *141*(10), 907-915.
79. Zhang, S. M., Lee, I. M., Manson, J. E., Cook, N. R., Willett, W. C., & Buring, J. E. (2007). Alcohol consumption and breast cancer risk in the Women's Health Study. *American Journal of Epidemiology*, *165*(6), 667-676.
80. Kim, H. J., Jung, S., Eliassen, A. H., Chen, W. Y., Willett, W. C., & Cho, E. (2017). Alcohol consumption and breast cancer risk in younger women according to family history of breast cancer and folate intake. *American journal of epidemiology*, *186*(5), 524-531.
81. Fuchs, C. S., Stampfer, M. J., Colditz, G. A., Giovannucci, E. L., Manson, J. E., Kawachi, I., ... & Willett, W. C. (1995). Alcohol consumption and mortality among women. *New England Journal of Medicine*, *332*(19), 1245-1250.
82. Sinnadurai, S., Okabayashi, S., Kawamura, T., Mori, M., Bhoo-Pathy, N., Taib, N. A., ... & JACC Study Group. (2020). Intake of common alcoholic and non-alcoholic beverages and breast cancer risk among Japanese women: Findings from the Japan collaborative cohort study. *Asian Pacific journal of cancer prevention: APJCP*, *21*(6), 1701.
83. Heberg, J., Simonsen, M. K., Danielsen, A. K., Klausen, T. W., Zoffmann, V., & Thomsen, T. (2019). Joint tobacco smoking and alcohol intake exacerbates cancer risk in women–the Danish nurse cohort. *European Journal of Oncology Nursing*, *43*, 101675.
84. Chen, W. Y., Rosner, B., Hankinson, S. E., Colditz, G. A., & Willett, W. C. (2011). Moderate alcohol consumption during adult life, drinking patterns, and breast cancer risk. *Jama*, *306*(17), 1884-1890.
85. Suzuki, R., Ye, W., Rylander-Rudqvist, T., Saji, S., Colditz, G. A., & Wolk, A. (2005). Alcohol and postmenopausal breast cancer risk defined by estrogen and progesterone receptor status: a prospective cohort study. *Journal of the National Cancer Institute*, *97*(21), 1601-1608.
86. Lew, J. Q., Freedman, N. D., Leitzmann, M. F., Brinton, L. A., Hoover, R. N., Hollenbeck, A. R., ... & Park, Y. (2009). Alcohol and risk of breast cancer by histologic type and hormone receptor status in postmenopausal women: the NIH-AARP Diet and Health Study. *American journal of epidemiology*, *170*(3), 308-317.
87. Rohan, T. E., Jain, M., Howe, G. R., & Miller, A. B. (2000). Alcohol consumption and risk of breast cancer: a cohort study. *Cancer Causes & Control*, *11*, 239-247.
88. Li, C. I., Chlebowski, R. T., Freiberg, M., Johnson, K. C., Kuller, L., Lane, D., ... & Prentice, R. (2010). Alcohol consumption and risk of postmenopausal breast cancer by subtype: the women's health initiative observational study. *Journal of the National Cancer Institute*, *102*(18), 1422-1431.
89. Simon, M. S., Carman, W., Wolfe, R., & Schottenfeld, D. (1991). Alcohol consumption and the risk of breast cancer: a report from the Tecumseh Community Health Study. *Journal of clinical epidemiology*, *44*(8), 755-761.
90. Garland, M., Hunter, D. J., Colditz, G. A., Spiegelman, D. L., Manson, J. E., Stampfer, M. J., & Willett, W. C. (1999). Alcohol consumption in relation to breast cancer risk in a cohort of United States women 25–42 years of age. *Cancer Epidemiology Biomarkers & Prevention*, *8*(11), 1017-1021.
91. Feigelson, H. S., Calle, E. E., Robertson, A. S., Wingo, P. A., & Thun, M. J. (2001). Alcohol consumption increases the risk of fatal breast cancer (United States). *Cancer Causes & Control*, *12*, 895-902.
92. Suzuki, R., Iwasaki, M., Inoue, M., Sasazuki, S., Sawada, N., Yamaji, T., ... & Tsugane, S. (2010). Alcohol consumption‐associated breast cancer incidence and potential effect modifiers: the Japan Public Health Center‐based Prospective Study. *International journal of cancer*, *127*(3), 685-695.
93. Mørch, L. S., Johansen, D., Thygesen, L. C., Tjønneland, A., Løkkegaard, E., Stahlberg, C., & Grønbæk, M. (2007). Alcohol drinking, consumption patterns and breast cancer among Danish nurses: a cohort study. *European journal of public health*, *17*(6), 624-629.
94. Petri, A. L., Tjønneland, A., Gamborg, M., Johansen, D., Høidrup, S., Sørensen, T. I., & Grønbæk, M. (2004). Alcohol intake, type of beverage, and risk of breast cancer in pre‐and postmenopausal women. *Alcoholism: Clinical and Experimental Research*, *28*(7), 1084-1090.
95. Feigelson, H. S., Jonas, C. R., Robertson, A. S., McCullough, M. L., Thun, M. J., & Calle, E. E. (2003). Alcohol, folate, methionine, and risk of incident breast cancer in the American Cancer Society Cancer Prevention Study II Nutrition Cohort. *Cancer Epidemiology Biomarkers & Prevention*, *12*(2), 161-164.
96. Baglietto, L., English, D. R., Gertig, D. M., Hopper, J. L., & Giles, G. G. (2005). Does dietary folate intake modify effect of alcohol consumption on breast cancer risk? Prospective cohort study. *Bmj*, *331*(7520), 807.
97. Horn-Ross, P. L., Hoggatt, K. J., West, D. W., Krone, M. R., Stewart, S. L., Anton-Culver, H., ... & Ziogas, A. (2002). Recent diet and breast cancer risk: the California Teachers Study (USA). *Cancer Causes & Control*, *13*, 407-415.
98. Høyer, A. P., & Engholm, G. (1992). Serum lipids and breast cancer risk: a cohort study of 5,207 Danish women. *Cancer Causes & Control*, *3*, 403-408.
99. Li, Y., Baer, D., Friedman, G. D., Udaltsova, N., Shim, V., & Klatsky, A. L. (2009). Wine, liquor, beer and risk of breast cancer in a large population. *European journal of cancer*, *45*(5), 843-850.
100. Thygesen, L. C., Mørch, L. S., Keiding, N., Johansen, C., & Grønbæk, M. (2008). Use of baseline and updated information on alcohol intake on risk for breast cancer: importance of latency. *International journal of epidemiology*, *37*(3), 669-677.
101. Falk, R. T., Maas, P., Schairer, C., Chatterjee, N., Mabie, J. E., Cunningham, C., ... & Ziegler, R. G. (2014). Alcohol and risk of breast cancer in postmenopausal women: an analysis of etiological heterogeneity by multiple tumor characteristics. *American journal of epidemiology*, *180*(7), 705-717.
102. Park, S. Y., Kolonel, L. N., Lim, U., White, K. K., Henderson, B. E., & Wilkens, L. R. (2014). Alcohol consumption and breast cancer risk among women from five ethnic groups with light to moderate intakes: the Multiethnic Cohort Study. *International journal of cancer*, *134*(6), 1504-1510.
103. Fagherazzi, G., Vilier, A., Boutron-Ruault, M. C., Mesrine, S., & Clavel-Chapelon, F. (2015). Alcohol consumption and breast cancer risk subtypes in the E3N-EPIC cohort. *European Journal of Cancer Prevention*, *24*(3), 209-214.
104. Shin, A., Sandin, S., Lof, M., Margolis, K. L., Kim, K., Couto, E., ... & Weiderpass, E. (2015). Alcohol consumption, body mass index and breast cancer risk by hormone receptor status: Women’Lifestyle and Health Study. *BMC cancer*, *15*(1), 1-6.
105. Chhim, A. S., Fassier, P., Latino-Martel, P., Druesne-Pecollo, N., Zelek, L., Duverger, L., ... & Touvier, M. (2015). Prospective association between alcohol intake and hormone-dependent cancer risk: modulation by dietary fiber intake. *The American Journal of Clinical Nutrition*, 102(1), 182-189.
