# Supplementary material for: Cancer risk based on alcohol consumption levels: a comprehensive systematic review and meta-analysis
Source: Epidemiol Health. 2023 Oct 16;45:e2023092. doi: 10.4178/epih.e2023092 (PMC10867516; doi:10.4178/epih.e2023092)
Supplement: Supplement Material 2. — Summary of confounding factors by cancer type in selected literature. [file epih-45-e2023092-Supplementary-2.docx]

Supplementary Material 2. Summary of confounding factors by cancer type in selected literature.

| Cancer types | Major considered confounders |
| --- | --- |
| Esophageal | age, sex, study area (or residential), BMI, smoking status, education |
| Stomach | age, sex, study area (or residential), BMI, smoking status, education |
| Liver | age, sex, study area (or residential), BMI, smoking status, education, diabetes, socioeconomic(income) status |
| Pancreatic | age, sex, study area (or residential), BMI, smoking status, education, physical activity, race/ethnicity |
| Colorectal | age, sex, family history, BMI, physical activity, smoking status, education, food or energy intake |
| Laryngeal | age, sex, study area (or residential), BMI, smoking status, physical activity |
| Lung | age, sex, study area (or residential), BMI, smoking status, education, physical activity, race/ethnicity, socioeconomic(income) status |
| Thyroid | age, sex, BMI, smoking status, education, race/ethnicity |
| Prostate | age, BMI, smoking status, education, race/ethnicity, family history, physical activity |
| Breast | age, family history, BMI, smoking status, education, physical activity, race/ethnicity, menopausal status, parity, oral contraceptive, use of hormone replacement therapy |

BMI, body mass index.

Variables evaluated as confounding factors were listed in at least one of the reviewed studies.
