# Supplementary material for: Cancer risk based on alcohol consumption levels: a comprehensive systematic review and meta-analysis
Source: Epidemiol Health. 2023 Oct 16;45:e2023092. doi: 10.4178/epih.e2023092 (PMC10867516; doi:10.4178/epih.e2023092)
Supplement: Supplement Material 3. — All cancer and sex specific pooled RR estimates by meta-analysis. [file epih-45-e2023092-Supplementary-3.docx]

Supplementary Material 3. All cancer and sex specific pooled RR estimates by meta-analysis.

|  | Alcohol consumption levels | | | | | | | | | | | |
| --- | --- | --- | --- | --- | --- | --- | --- | --- | --- | --- | --- | --- |
| Sex | Light | | | Light to moderate | | | Moderate to heavy | | | Heavy | | |
|  | No. | RR (95% CI) | *I^2^* | No. | RR (95% CI) | *I^2^* | No. | RR (95% CI) | *I^2^* | No. | RR (95% CI) | *I^2^* |
| Men | 30 | 1.05 (0.97-1.14) | 70% | 26 | 1.10 (1.02-1.20) | 67% | 21 | 1.33 (1.16-1.52) | 87% | 32 | 1.65 (1.43-1.91) | 84% |
| Women | 22 | 0.97 (0.95-1.00) | 46% | 16 | 0.98 (0.88-1.08) | 59% | 9 | 1.06 (1.00-1.11) | 25% | 10 | 1.08 (0.88-1.33) | 52% |

The range of alcohol consumption levels was divided into light (0.01–12.4 g/day), light to moderate (12.5–24.9 g/day), moderate to heavy (25.0–49.9 g/day), and heavy (50.0+ g/day). RR, relative risk; 95% CI, 95% confidence intervals; *I^2^*, indicates heterogeneity.
