# Supplementary material for: Cancer risk based on alcohol consumption levels: a comprehensive systematic review and meta-analysis
Source: Epidemiol Health. 2023 Oct 16;45:e2023092. doi: 10.4178/epih.e2023092 (PMC10867516; doi:10.4178/epih.e2023092)
Supplement: Supplement Material 4. — Funnel plots of the meta-analysis of studies examining the relationship between all cancer and alcohol consumption levels. [file epih-45-e2023092-Supplementary-4.docx]

Supplementary Material 4. Funnel plots of the meta-analysis of studies examining the relationship between all cancer and alcohol consumption levels.

| Light | Light to moderate |
| --- | --- |
| 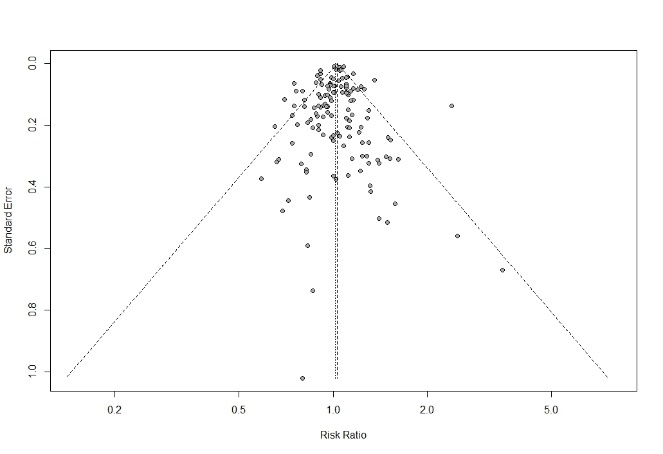 | 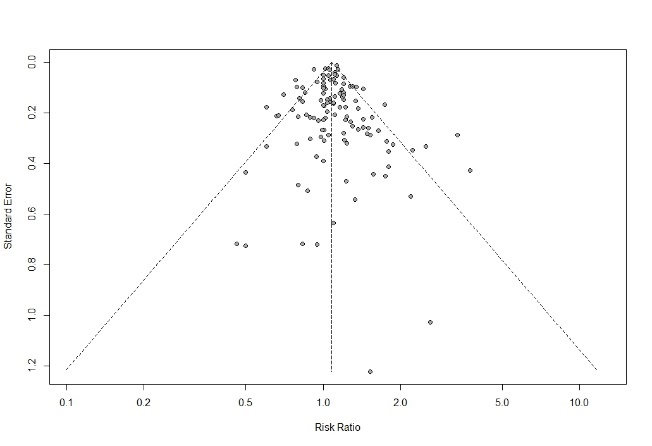 |
| Egger test *p*-value = 0.42 | Egger test *p*-value = 0.56 |
| Moderate to heavy | Heavy |
| 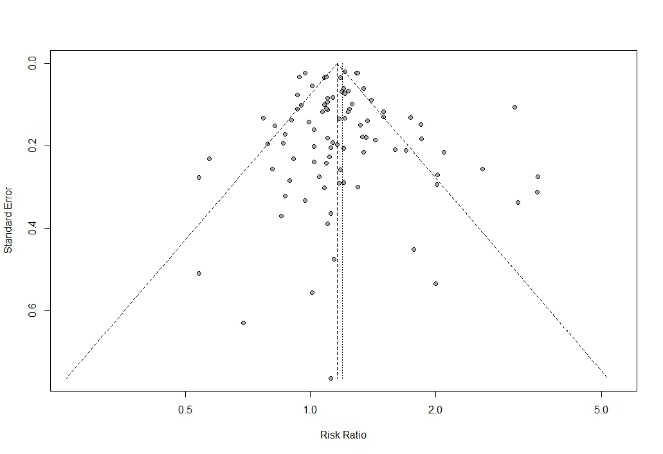 | 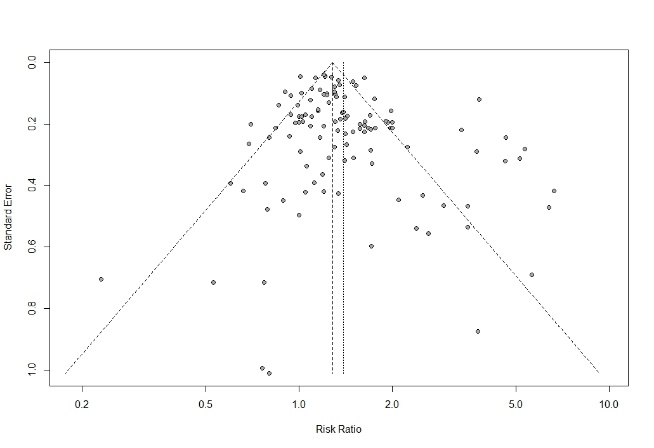 |
| Egger test *p*-value = 0.39 | Egger test *p*-value = 0.01 |

The range of alcohol consumption levels was divided into light (0.01–12.4 g/day), light to moderate (12.5–24.9 g/day), moderate to heavy (25.0–49.9 g/day), and heavy (50.0+ g/day). RR, relative risk; 95% CI, 95% confidence intervals.

^a^ All cancer (esophageal cancer; stomach cancer; liver cancer; pancreatic cancer; colorectal cancer; laryngeal cancer; lung cancer; prostate cancer; breast cancer; thyroid cancer).
