# Supplementary material for: Cancer risk based on alcohol consumption levels: a comprehensive systematic review and meta-analysis
Source: Epidemiol Health. 2023 Oct 16;45:e2023092. doi: 10.4178/epih.e2023092 (PMC10867516; doi:10.4178/epih.e2023092)
Supplement: Supplement Material 5. — Funnel plots of the meta-analysis of studies examining the relationship between cancer type and alcohol consumption levels. [file epih-45-e2023092-Supplementary-5.docx]

Supplementary Material 5. Funnel plots of the meta-analysis of studies examining the relationship between cancer type and alcohol consumption levels.

A. Esophageal cancer

| Light | Light to moderate |
| --- | --- |
| 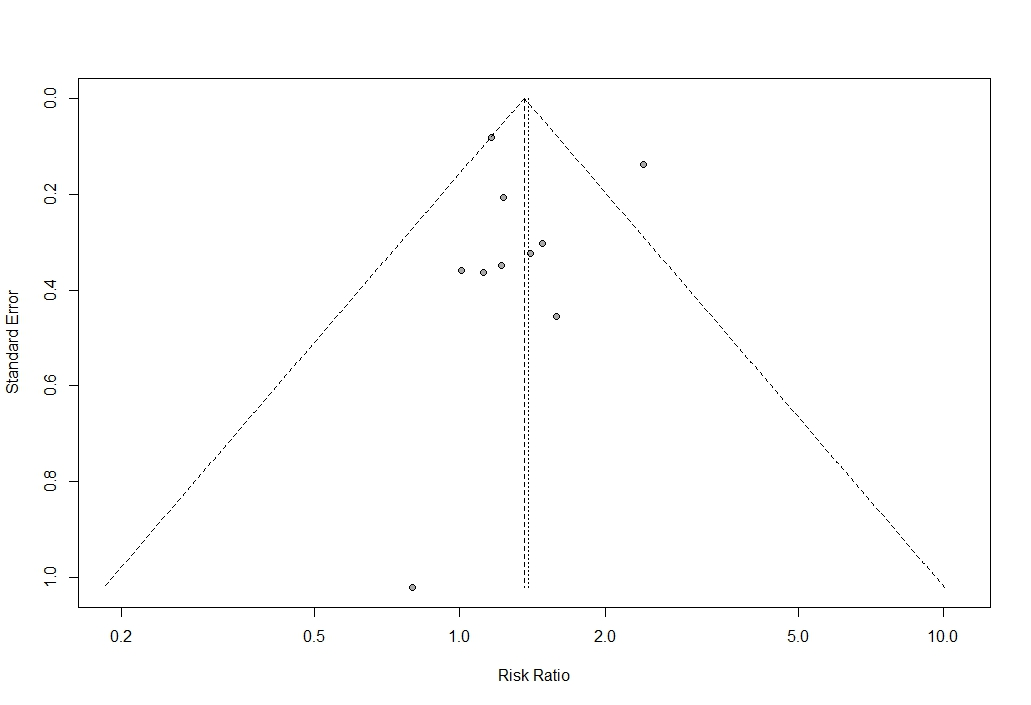 | 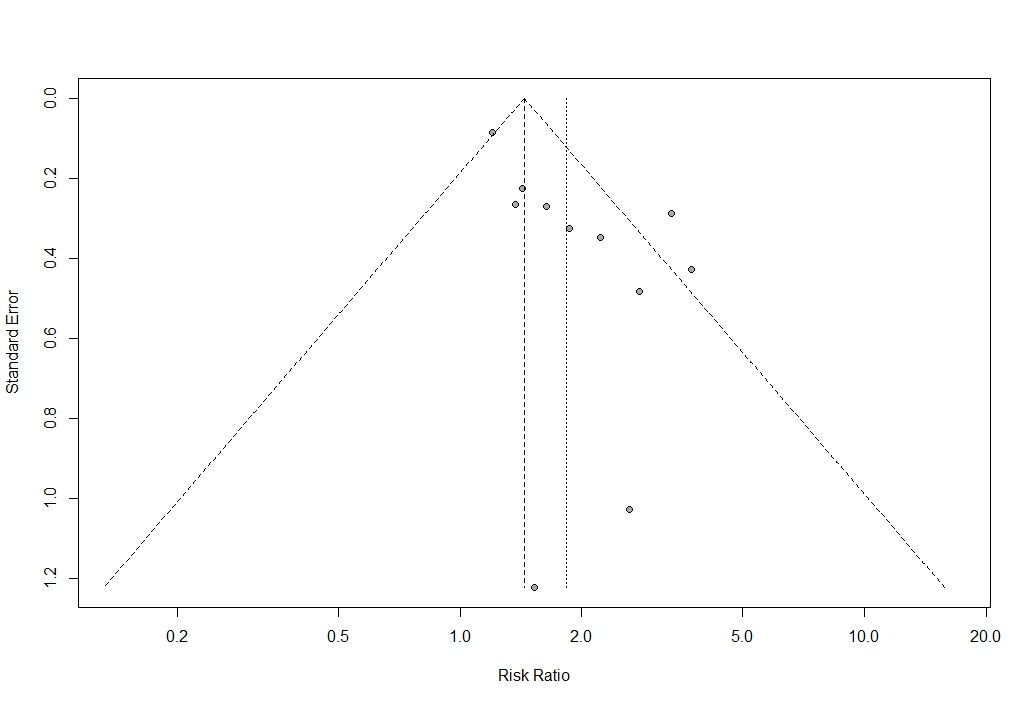 |
| Egger test *p*-value = 0.95 | Egger test *p*-value = 0.01 |
| Moderate to heavy | Heavy |
| - | 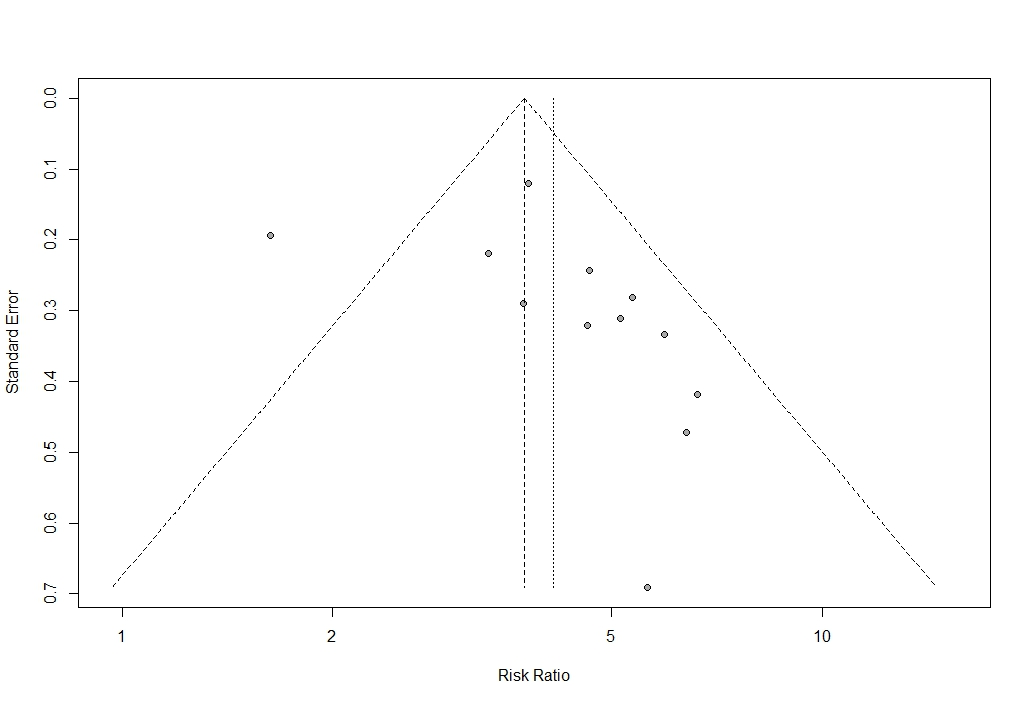 |
| - | Egger test *p*-value = 0.14 |

The range of alcohol consumption levels was divided light (0.01–12.4 g/day), light to moderate (12.5–24.9 g/day), moderate to heavy (25.0–49.9 g/day), and heavy (50.0+ g/day).

B. Colorectal cancer

| Light | Light to moderate |
| --- | --- |
| 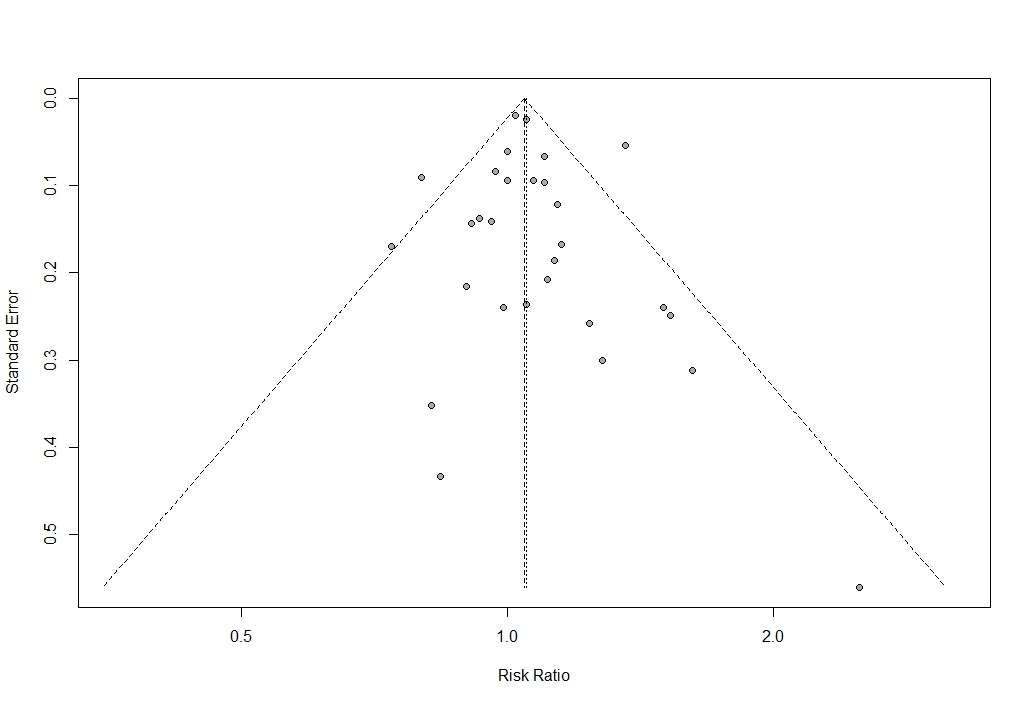 | 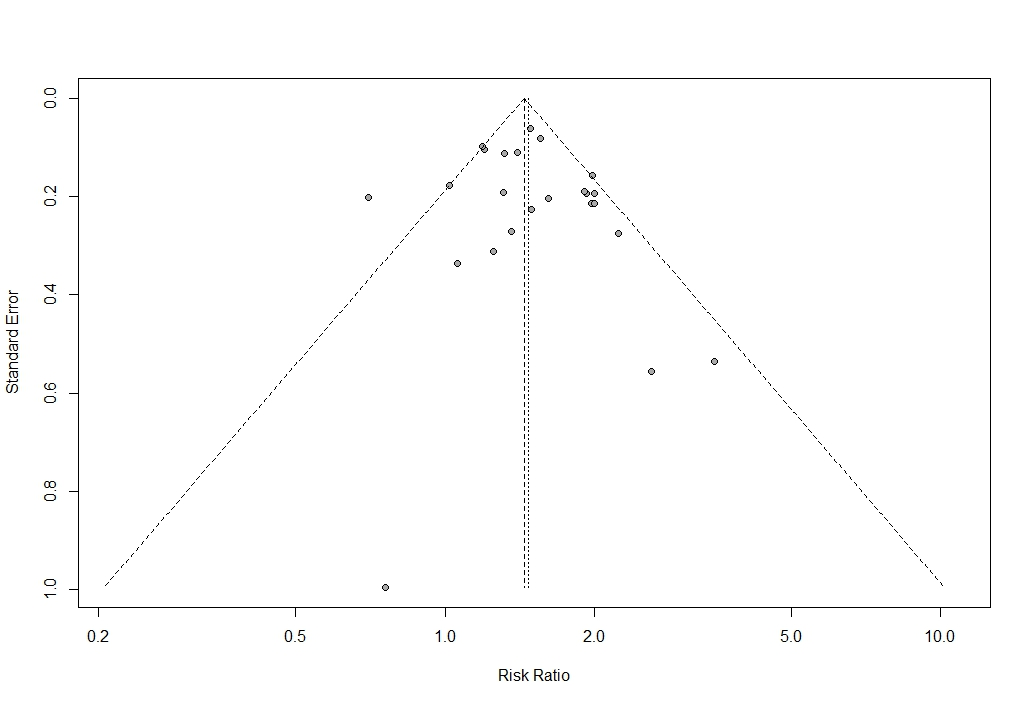 |
| Egger test *p*-value = 0.57 | Egger test *p*-value = 0.75 |
| Moderate to heavy | Heavy |
| 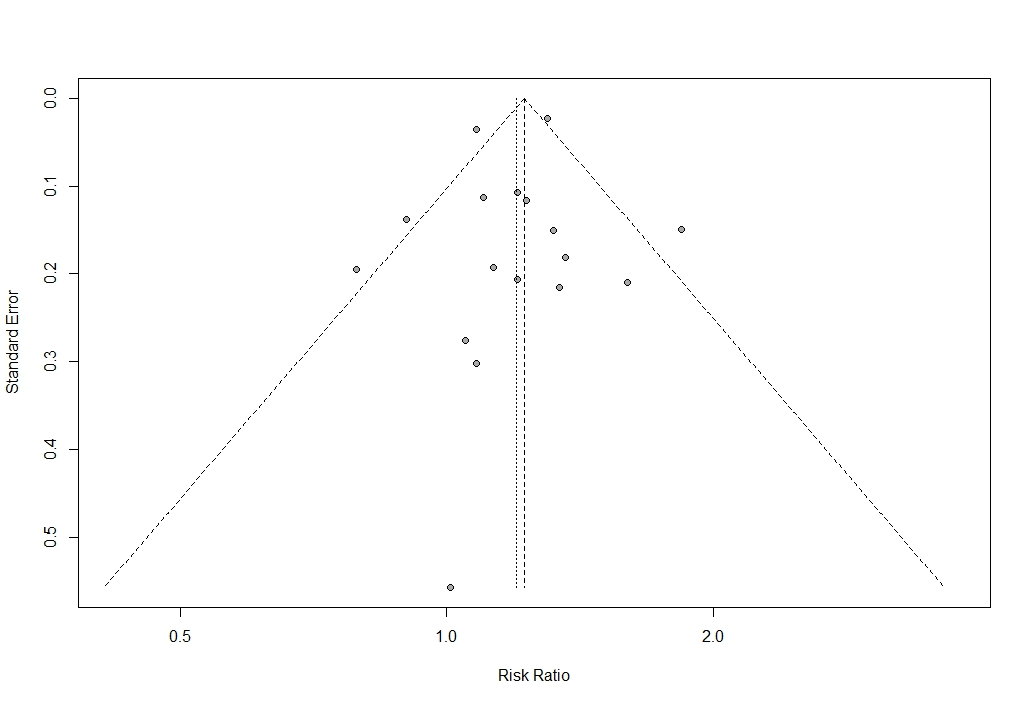 | 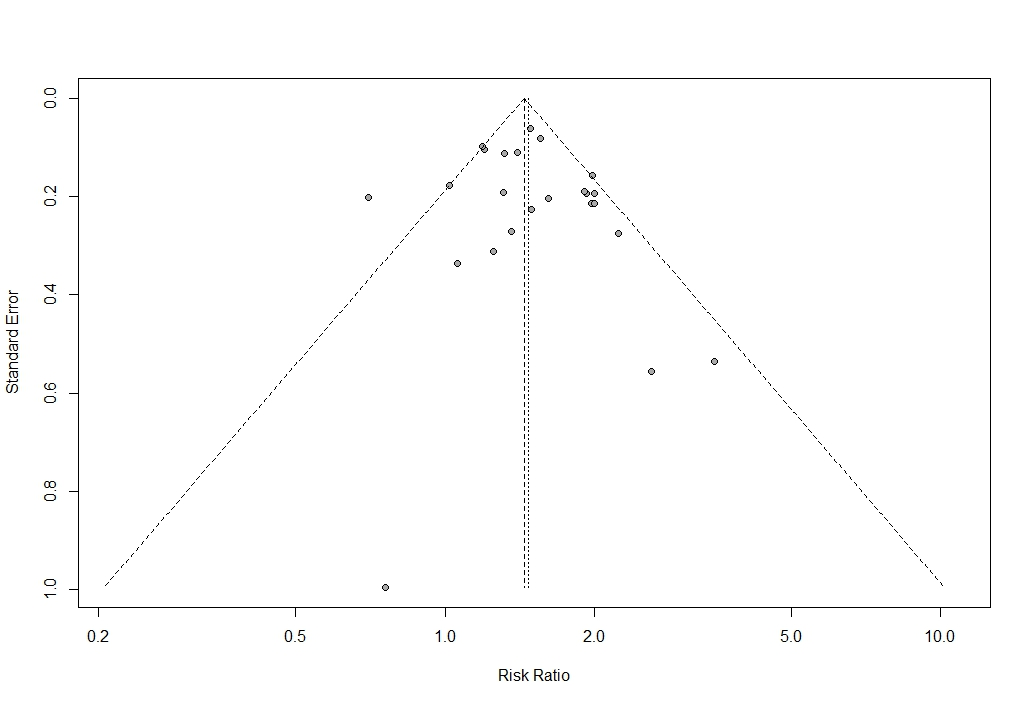 |
| Egger test *p*-value = 0.59 | Egger test *p*-value = 0.89 |

The range of alcohol consumption levels was divided into light (0.01–12.4 g/day), light to moderate (12.5–24.9 g/day), moderate to heavy (25.0–49.9 g/day), and heavy (50.0+ g/day).

C. Lung cancer

| Light | Light to moderate |
| --- | --- |
| 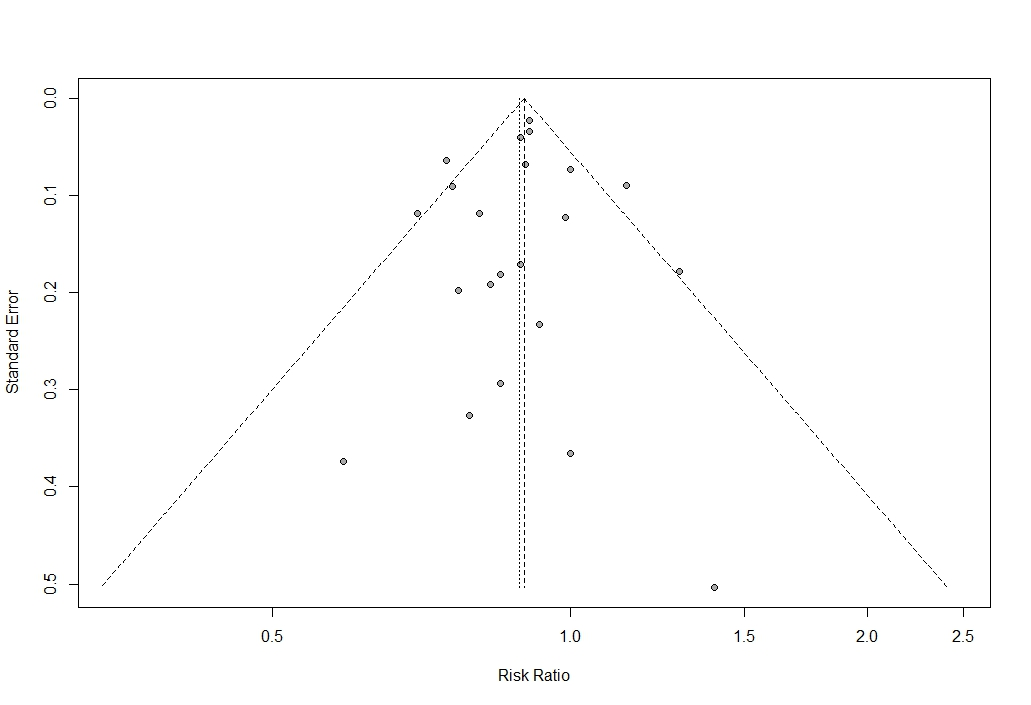 | 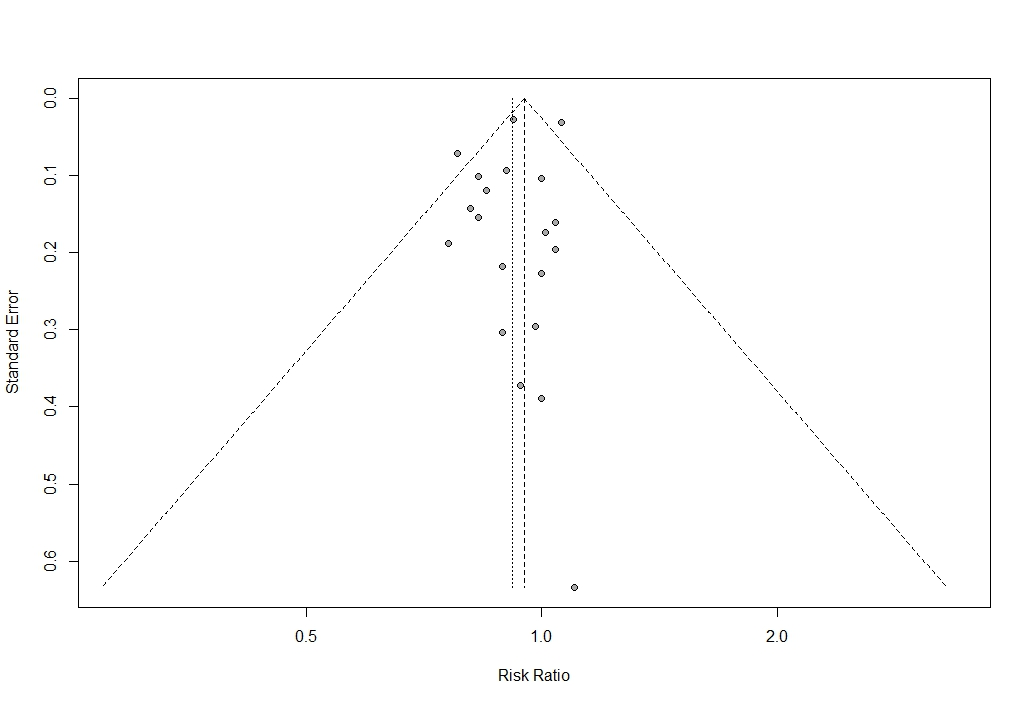 |
| Egger test *p*-value = 0.66 | Egger test *p*-value = 0.28 |
| Moderate to heavy | Heavy |
| 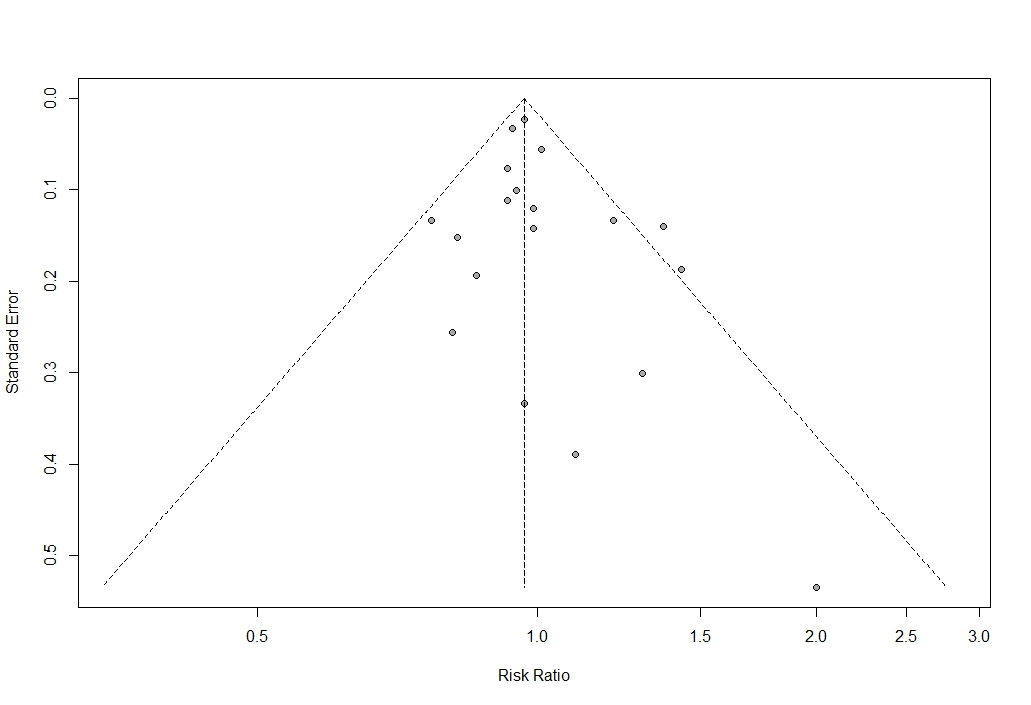 | 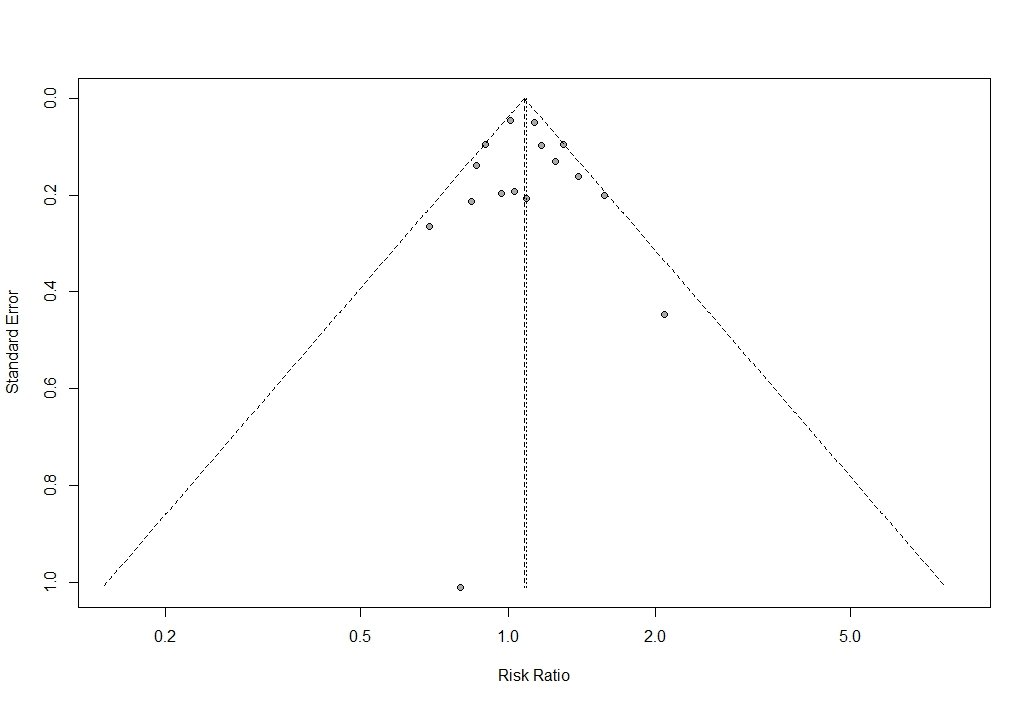 |
| Egger test *p*-value = 0.27 | Egger test *p*-value = 0.78 |

The range of alcohol consumption levels was divided into light (0.01–12.4 g/day), light to moderate (12.5–24.9 g/day), moderate to heavy (25.0–49.9 g/day), and heavy (50.0+ g/day).

D. Prostate cancer

| Light | Light to moderate |
| --- | --- |
| 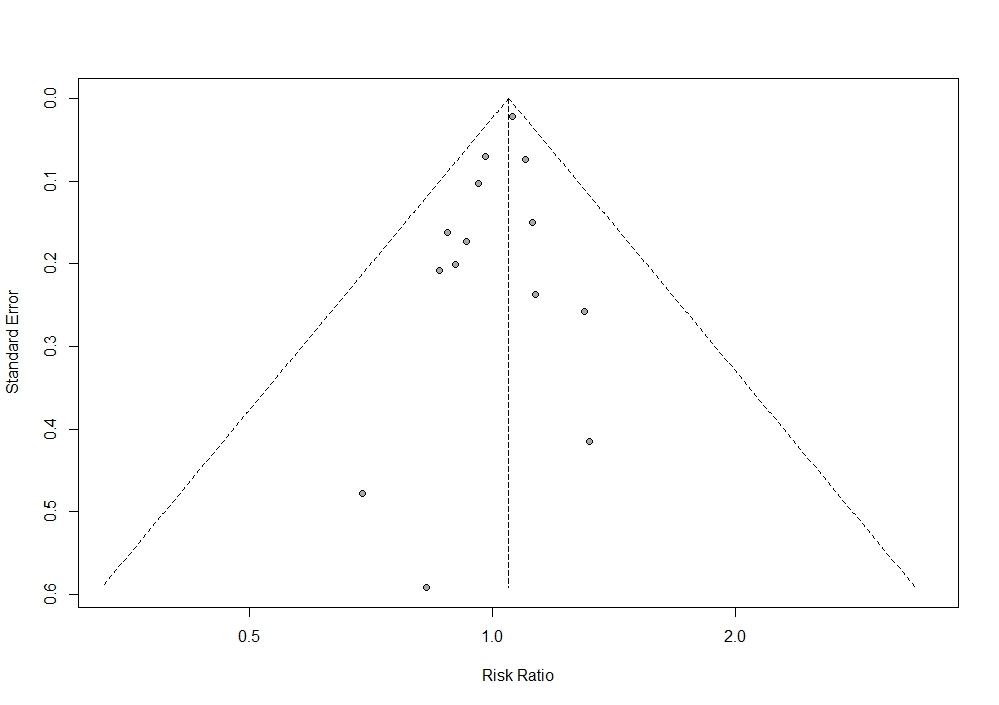 | 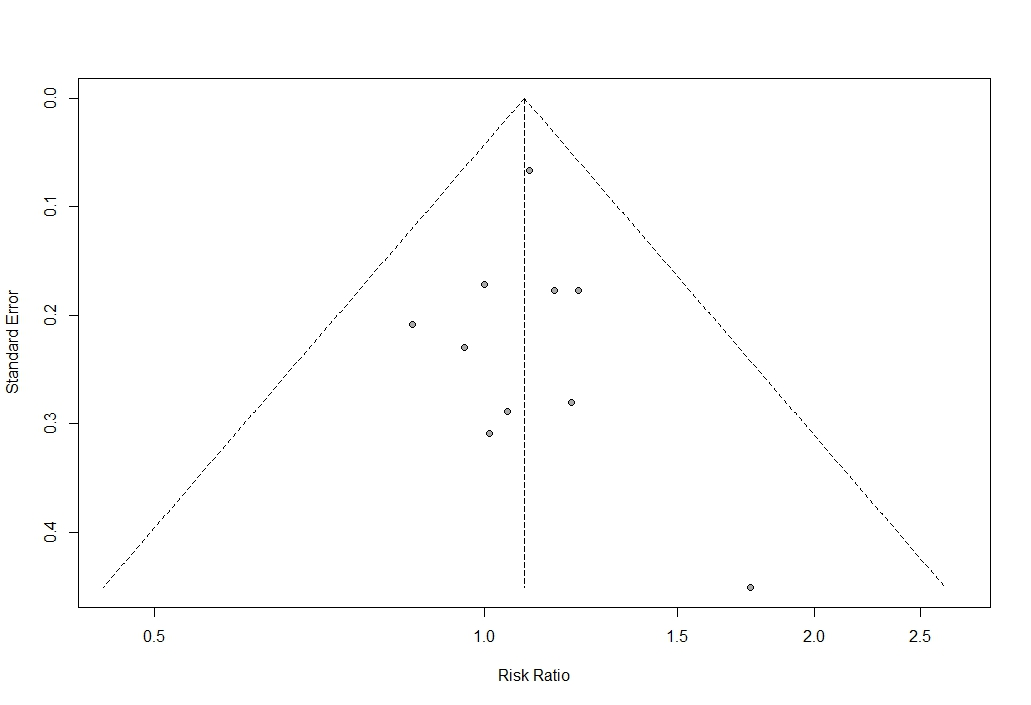 |
| Egger test *p*-value = 0.16 | Egger test *p*-value = 0.96 |
| Moderate to heavy | Heavy |
| 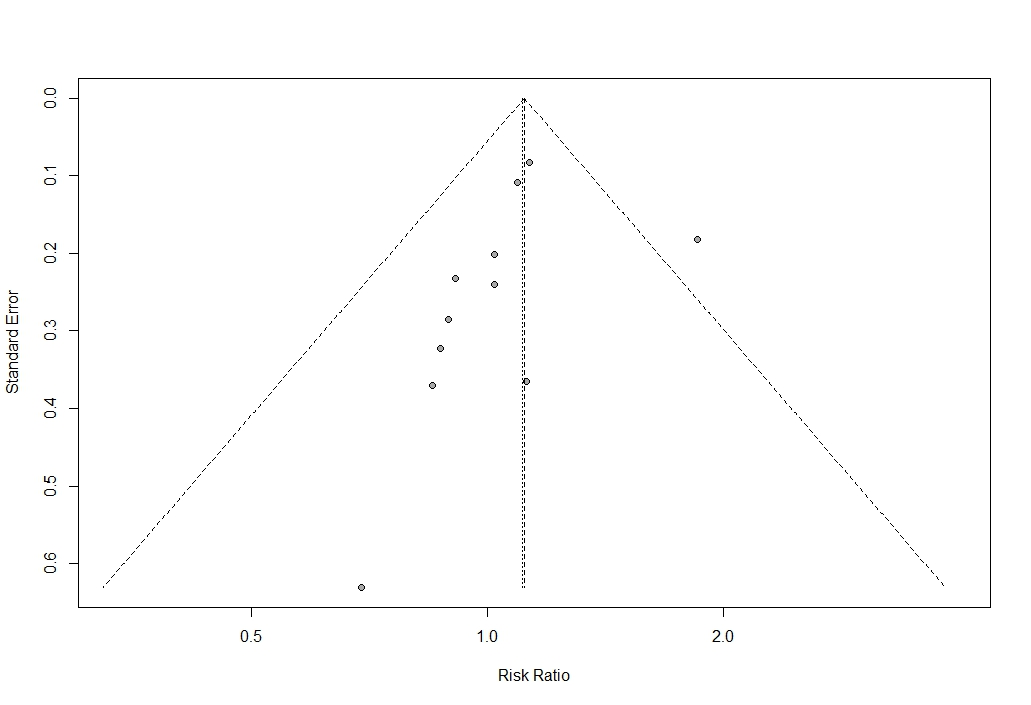 | 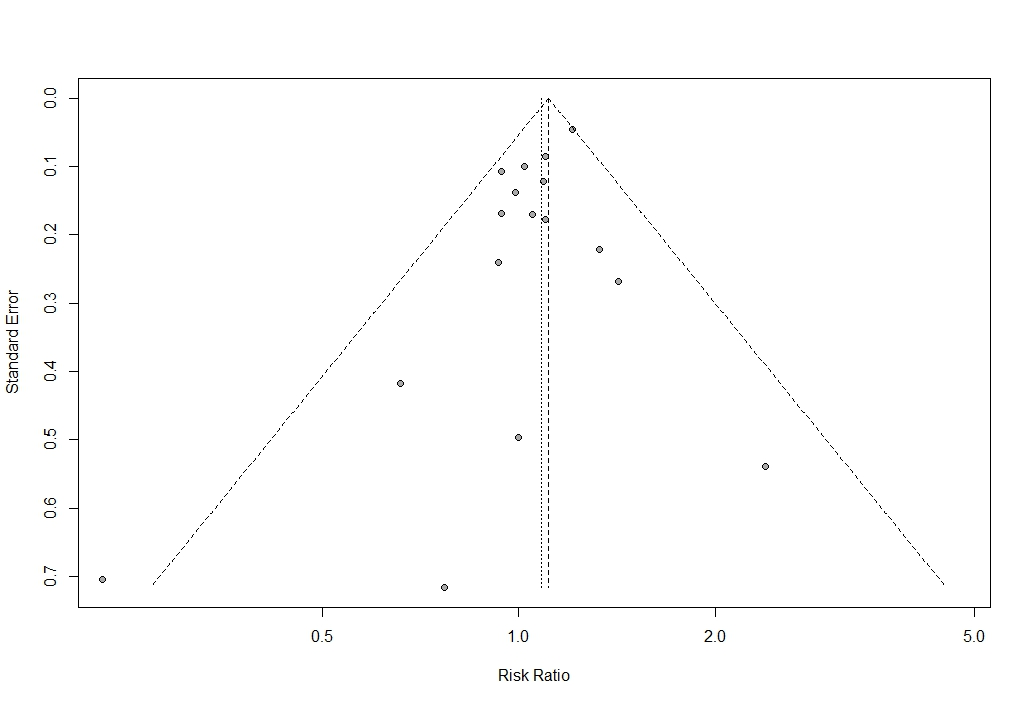 |
| Egger test *p*-value = 0.32 | Egger test *p*-value = 0.08 |

The range of alcohol consumption levels was divided into light (0.01–12.4 g/day), light to moderate (12.5–24.9 g/day), moderate to heavy (25.0–49.9 g/day), and heavy (50.0+ g/day).

E. Breast cancer

| Light | Light to moderate |
| --- | --- |
| 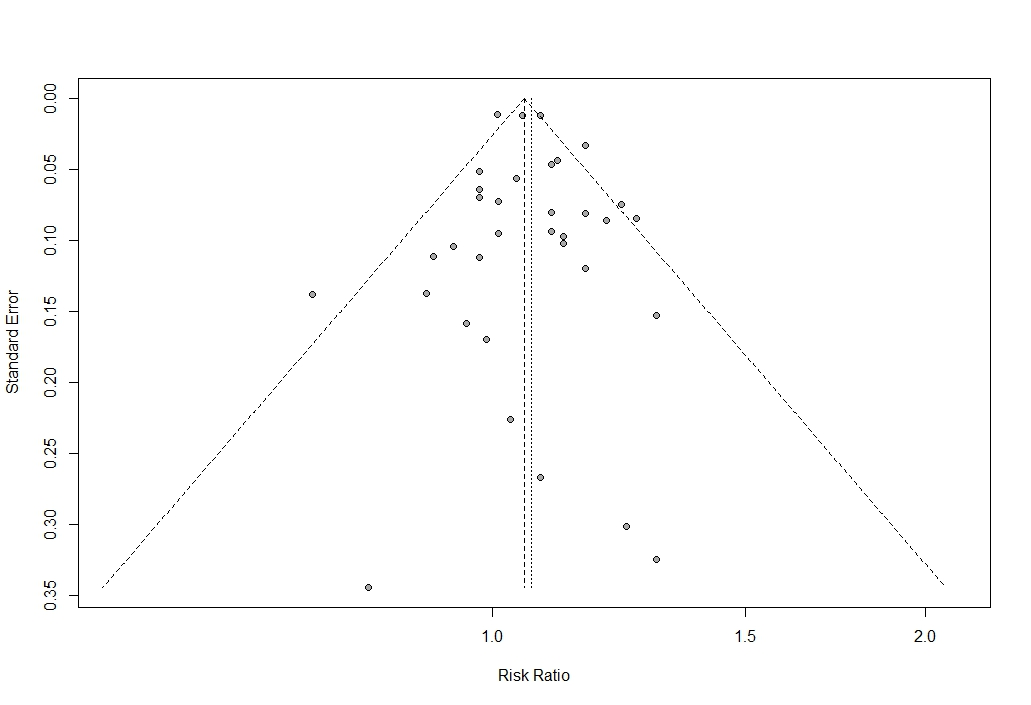 | 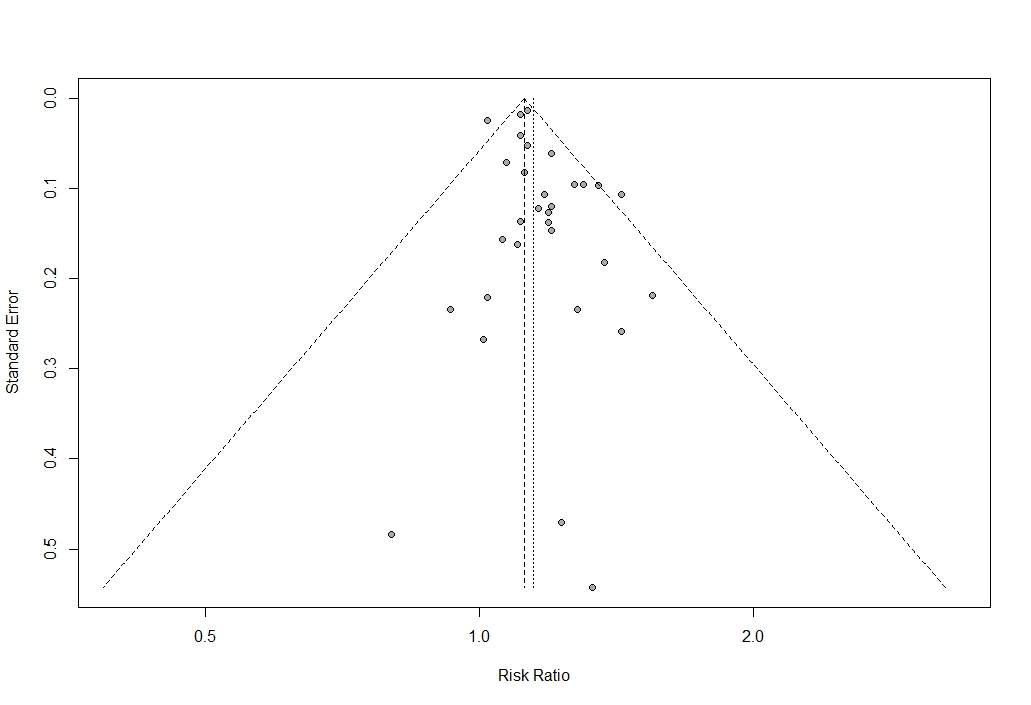 |
| Egger test *p*-value = 0.53 | Egger test *p*-value = 0.04 |
| Moderate to heavy | Heavy |
| 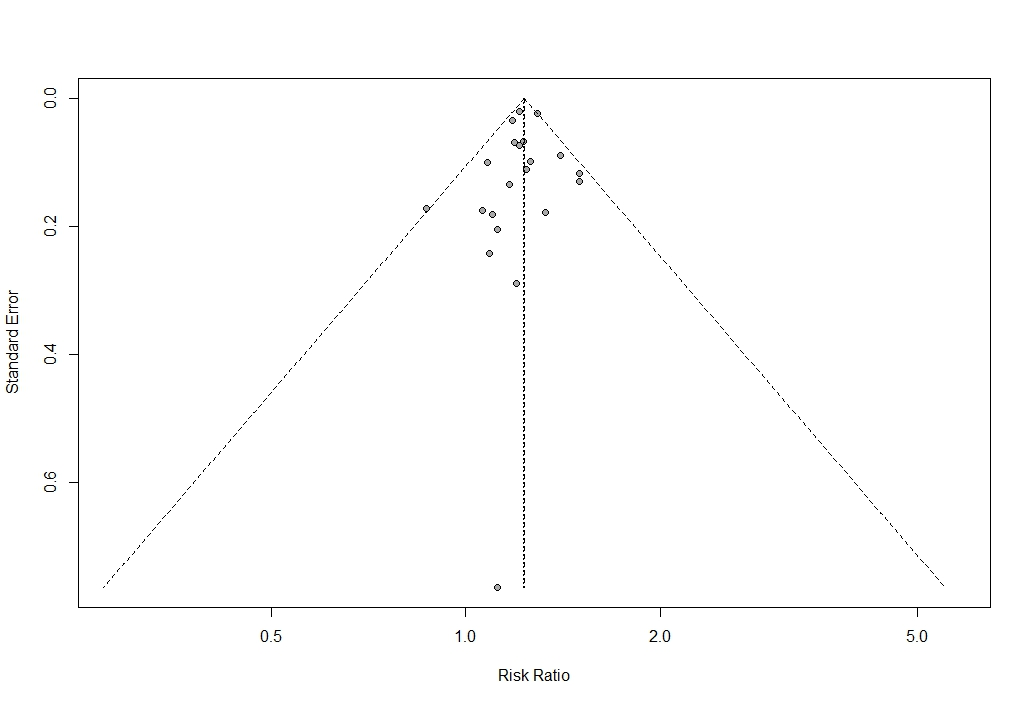 | 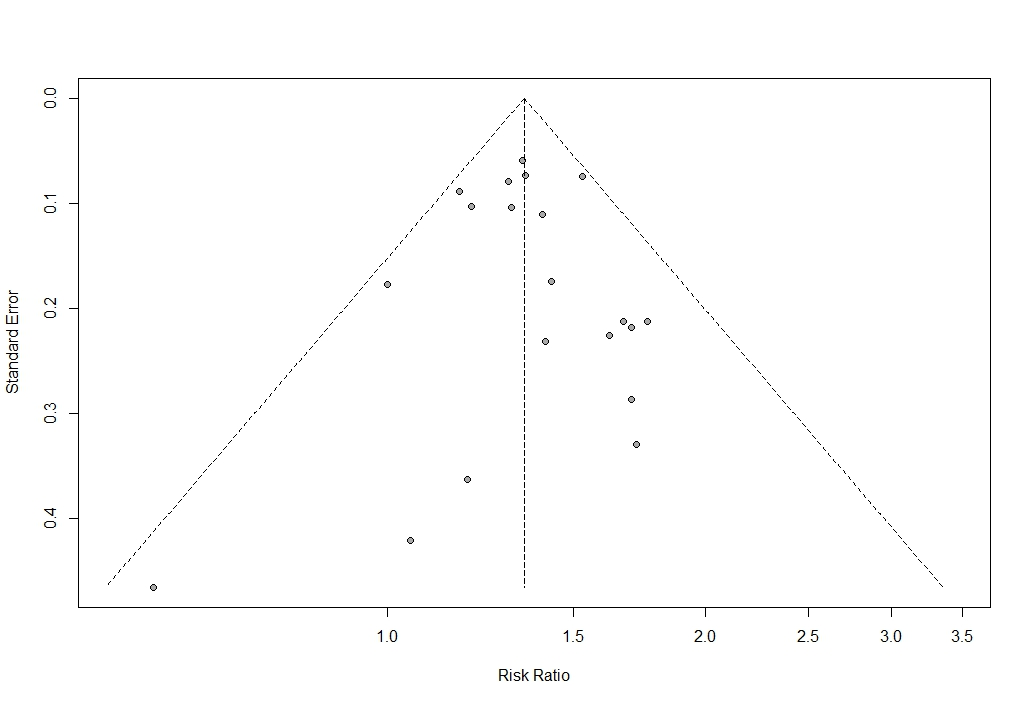 |
| Egger test *p*-value = 0.56 | Egger test *p*-value = 0.83 |

The range of alcohol consumption levels was divided into light (0.01–12.4 g/day), light to moderate (12.5–24.9 g/day), moderate to heavy (25.0–49.9 g/day), and heavy (50.0+ g/day).
